# Incriminating vectors of deer malaria (*Plasmodium odocoilei*) at a Florida deer farm

**DOI:** 10.1186/s13071-025-06942-5

**Published:** 2025-07-28

**Authors:** Morgan Rockwell, Samantha M. Wisely, Derrick K. Mathias, Nathan D. Burkett-Cadena

**Affiliations:** 1 Florida Medical Entomology Laboratory, IFAS, University of Florida, 200 9thSt. SE, Vero Beach, FL 32962 USA; 2 Wildlife Ecology and Conservation Department, University of Florida, Gainesville, FL USA

**Keywords:** Vector-borne disease, *Plasmodium*, *Anopheles*, Malaria, White-tailed deer

## Abstract

**Background:**

*Plasmodium odocoilei*, the only nonhuman *Plasmodium* parasite of native mammals in North America, infects white-tailed deer (WTD) throughout the eastern USA. Although deer malaria is not a significant cause of disease in healthy deer, infection with *P. odocoilei* may increase susceptibility to infection with and mortality due to epizootic hemorrhagic disease virus in deer fawns. The incrimination of the vector(s) of deer malaria is an essential step in developing management plans for reducing the incidence of deer malaria.

**Methods:**

At a deer farm in Gadsden County, FL, with previously documented evidence of deer malaria transmission, mosquitoes were collected using carbon-dioxide-baited light traps, aspirators, and resting shelters. White-tailed deer host use and *P. odocoilei* infection rates were quantified in potential vector mosquito samples using polymerase chain reaction and Sanger sequencing.

**Results:**

Diverse mosquito species (*n* = 38) were active at the deer farm. Four mosquito species or species complexes specialized in feeding on WTD were observed, taking at least 75% of blood meals from this one host species: *Anopheles quadrimaculatus* s.l. (88.9%), *Anopheles punctipennis* (83.3%), *Anopheles crucians* s.l. (81.4%), and *Culex erraticus* (87.7%). The highest infection rate of *P. odocoilei* was found in *An. quadrimaculatus* s.l. (4.1%), followed by *An. punctipennis* (3.1%), and *An. crucians* s.l. (0.47%). No other mosquito species were found to be infected with *P. odocoilei*.

**Conclusions:**

*Anopheles quadrimaculatus* s.l., *An. punctipennis*, and *An. crucians* s.l. met three of the criteria for vector incrimination. These species were present in areas inhabited by WTD, specialized in feeding on WTD, and were naturally infected with *P. odocoilei*. *Anopheles quadrimaculatus* s.l. and *Anopheles punctipennis* are the most likely natural vectors of deer malaria in Florida, as indicated by their high percentages of WTD blood meals (> 83%) and relatively high infection rates (> 3%). To fully incriminate the vector(s) for *P. odocoilei*, laboratory vector competence studies are needed to determine their ability to biologically transmit the parasites.

**Graphical Abstract:**

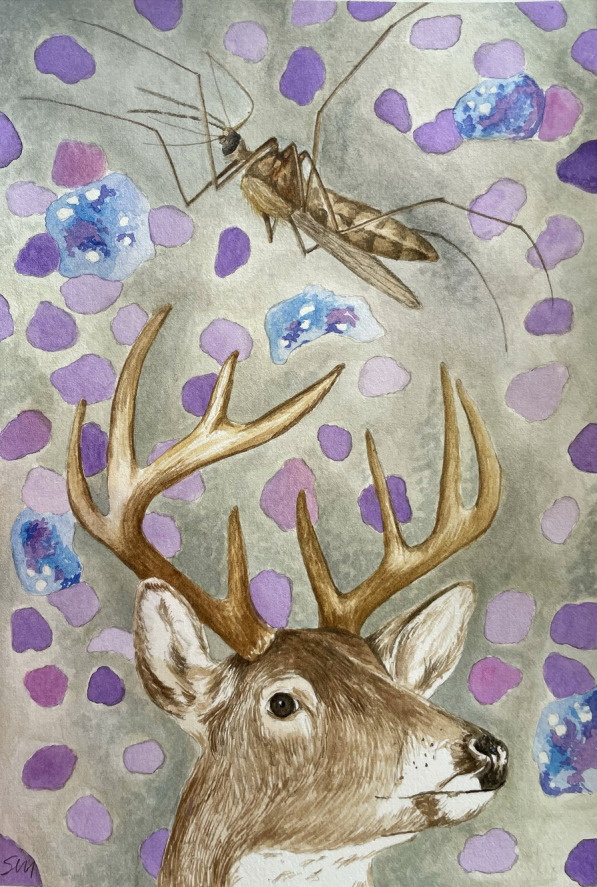

**Supplementary information:**

The online version contains supplementary material available at 10.1186/s13071-025-06942-5.

## Background

Deer farming is an important industry in the USA, with annual revenue reaching up to USD 7.9 billion [1]. However, deer farming has been heavily impacted by various vector-borne diseases [2–4]. Deer malaria is the only malaria affecting nonhuman mammals in North America [5, 6]. It is caused by *Plasmodium odocoilei*, member of a genus of protozoans that infects the red blood cells of their vertebrate hosts and is transmitted primarily by mosquitoes [7]. A survey of several ungulate species across the USA found that *P. odocoilei* only infects *Odocoileus virginianus*, white-tailed deer (WTD), which is the most widely distributed wild ungulate in North America [8].

The impacts of *P. odocoilei* on WTD health are poorly understood, but the parasite likely causes subclinical disease [2] and may also increase susceptibility to infection with arboviruses and arbovirus-induced mortality [2, 8]. *Plasmodium odocoilei* was discovered and then described from the blood of a splenectomized WTD in Texas, USA, during a survey of hemosporidian parasites in deer in 1967 [5, 6]. Some general signs of disease were noted, although they were not definitively attributable to deer malaria [2]. Deer malaria was not reported again until it was detected by researchers investigating transmission ecology of avian malaria in 2016 [9]. During that study, an unidentified malaria parasite was detected in a blood-engorged female of *Anopheles punctipennis*, a species of mosquito that rarely bites birds [10–14]. Polymerase chain reaction (PCR) and sequencing determined that the *An. punctipennis* female fed upon a WTD, leading them to suspect and then confirm that the parasite was *P. odocoilei* [9]. By screening diverse ungulates across the USA, the investigators found a high infection rate of *Plasmodium* in WTD across the eastern USA (25%, 77/308), but not in the western USA or other ungulate species [9]. Although the direct effects of *P. odocoilei* on WTD health are rarely reported, a study conducted on a deer farm in northern Florida demonstrated that a substantial percentage of fawns acquired *P. odocoilei* during their first 8 months of life (21%, 7/33) and documented a potential association between *P. odocoilei* infection and decreased fawn survival [8]. The study also found a higher infection rate of epizootic hemorrhagic disease virus (EHDV) in fawns infected with *Plasmodium* (86%, 6/7) compared with fawns that were not infected with *P. odocoilei* (50%, 13/26). These findings suggested that infection with *P. odocoilei* may increase the risk of acquiring EHDV and potentially other arboviruses [8].

The vector of *P. odocoilei* must be incriminated to understand the transmission of the parasite and to develop integrated pest management (IPM) strategies focused on the vector species. The following criteria must be fulfilled before a putative vector species can be incriminated as the vector(s) of *P. odocoilei* with reasonable certainty: (1) a spatial and temporal relationship between putative vector species and the host must correlate, (2) the vector must be in adequate numbers to transmit the parasite, (3) the putative vector species must be associated with feeding on the host carrying the pathogen, (4) the vector species must be naturally infected with the parasite in the wild, and (5) the putative vector species must be capable of experimentally transmitting the parasite between hosts in laboratory settings [15–18].

In this study, our goal was to partially incriminate vectors of deer malaria, focusing on the presence, host use, and *P. odocoilei* infection in mosquito species at a deer farm with documented transmission of deer malaria in northern Florida [8]. We determined which mosquito species specialize in feeding on WTD using PCR-based blood meal analysis and Sanger sequencing [19, 20]. We also compared the *P. odocoilei* infection rates among mosquito species using a nested PCR-assay. These data are essential to incriminate the vector(s) of deer malaria.

## Methods

### Sampling sites

Mosquitoes were sampled from a northern Florida deer farm in Gadsden County, which is used for breeding and hunting WTD and other cervid and bovine game species [21–23]. The 200 ha property included ten high-fenced pens with improved pastures (8.5 ha), which housed approximately 100 WTD, at a density of approximately 12 WTD/ha. Sick and injured WTD were housed in the “barn pens,” while healthy WTD used for breeding were kept in the “breeding pens.” Free-ranging WTD and other ruminants were located within the preserve, which encompassed the surrounding Gulf Coast Forest and contained around 130–150 WTD (0.74/ha). The landscape of the property consisted of pastures, wetlands, and mixed deciduous/coniferous forests [21, 23, 24]. The farm is fully characterized, including maps, in prior studies [21, 23].

### Mosquito sampling, identification, and processing

A variety of sampling methods were employed, including pop-up resting shelters, a large-diameter aspirator, a small-diameter aspirator, and carbon-dioxide-baited Centers for Disease Control and Prevention (CDC) miniature light traps (Model 2836BQ, BioQuip, Rancho Dominguez, CA, USA) to collect a diverse assemblage of mosquito species of different physiological statuses, including blood-engorged and nonengorged females [25]. Mosquitoes were collected daily during each of eight collection trips that lasted for 5 days each (October 2022; May, July, August, September, and October 2023; May and June 2024). For each day, all three collection methods were used at each area (breeding pens, barn pens, and the preserve).

Five to eight resting shelter traps were sampled per area. Resting shelters were set up the day prior to sampling, in shaded areas, with the opening toward the west [25]. Traps were sampled in the morning (8:30–10:30 a.m.), as female blood-engorged mosquitoes often seek refuge, often in cavities of dark, cool places, to digest their blood meal [26, 27]. The large-diameter aspirator consisted of galvanized air duct (20.3 cm in diameter, 61 cm long) with an electric radiator fan 20 cm in diameter powered by a rechargeable 12 V/6Ah gel-sealed battery [24]. The aspirator drew mosquitoes toward the collection cup, which was a screen-bottom 3.7 L plastic food storage container (Rubbermaid, Atlanta, GA, USA). For each site, the large-diameter aspirator was used three times daily for 2-min sessions in the morning (08:30–10:30) within a 200 m radius of the CDC light trap and in low vegetation (grasses, shrubs, and fallen limbs) [28]. The small diameter aspirator consisted of a 10 cm computer fan, attached to a clear plastic tube (120 mm in diameter), powered by a rechargeable 12 V/6Ah gel-sealed battery [25]. The small-diameter aspirator was operated for three 2-min sessions in the morning (8:30–10:30 a.m.) around crevices, burrows, and hollowed trees [25]. At each site, one CDC light trap baited with carbon dioxide was set at dusk (6:30–8:30 p.m.) and collected in the morning (8:30–10:30 a.m.). The light traps were hung above the ground from a 1.21 m shepherd hook. Each light trap was baited with dry ice in an insulated thermos canister that delivered carbon dioxide to the trap through its downward-directed spout. The traps were powered by rechargeable 6 V/12Ah gel-sealed batteries (Model SLAA6-12F, Duracell Ultra, Bethel, Connecticut, USA) [27].

Mosquitoes were identified to species on the basis of morphology using dichotomous keys in Darsie and Ward (2005) and Burkett–Cadena (2013) [29, 30]. Females belonging to the *Anopheles quadrimaculatus* subgroup (five species) and *Anopheles crucians* complex (seven species) cannot be reliably differentiated morphologically, and were recorded as *An. quadrimaculatus* s.l. and *An. crucians* s.l.

The abdomen of each blood-engorged female was separated from the head–thorax segments using a sterile micropipette tip. The blood meal was smeared onto a Whatman FTA Classic Card to preserve for subsequent blood meal analysis [31, 32]. Up to 25 nonengorged females and head–thorax segments of blood-engorged females of the same species were pooled and processed using nested PCR to detect *Plasmodium*.

For any thoracic segment that tested positive for *P. odocoilei*, the corresponding blood-engorged abdomen was also screened for *Plasmodium* to determine the developmental stage of the parasite. Detection of *Plasmodium* in thoracic segments may indicate the presence of infectious sporozoites in the salivary glands [18], whereas positive abdominal segments suggest the presence of either midgut oocysts or *Plasmodium*-infected erythrocytes derived from the host deer.

### Molecular analysis

DNA was extracted from samples using the InstaGene matrix (Biorad, Hercules, CA), following published protocols [31]. For identifying host species, vertebrate host DNA was amplified from the mosquito blood meal using the primer pair H2714 and L2513 (Table 1) targeting mitochondrial *16S rRNA* and *cytochrome* *b* gene (*cytb*) of mammals and amphibians [22, 27, 33]. Samples that did not amplify were then processed using the L0 and H1, then L0 and H0 primer pair targeting *cytb* gene of birds, and then 16L1 and H3056 primer pair targeting the *16S ribosomal RNA* (rRNA) of reptiles (Table 1) [34, 35]. A nested PCR assay was used to screen for the presence of *P. odocoilei* by amplification of *Plasmodium* DNA using a *cytb* gene [9]. A total of 2.5 µL of the resulting DNA was then used as a template for 25 µL amplification reactions. PCR amplifications were conducted in a solution containing 12.5 µL Invitrogen Platinum Green Hot Start PCR Master Mix, 0.5 µL of each primer (20 µM), 2.5 µL of DNA template, and 9 µL molecular-grade water. The nested PCR used the amplicon from an initial reaction as a template for the second reaction [36–38]. Table 1 provides primer sequences, target genes, expected amplicon sizes, and cycling conditions for all primers used.

**Table 1:** The primers that were used and their gene targets, sequences, expected amplicon size, and PCR cycle conditions

Primer name	Target gene	Sequence	Amplicon size (bp)	Cycle conditions*
H2714/L2513	*16S rRNA*	F: 5’-CTCCATAGGGTCTTCTCGTCTT-3’ R: 5’-GCCTGTTTACCAAAAACATCAC-3’	200	95/240, 95/30 (35), 57/30, 72/30, 72/420
16L1/H3056	*16S rRNA*	F: 5’-CTGACCGTGCAAAGGTAGCGTAATCACT-3’ R: 5’-CTCCGGTCTGAACTCAGATCACGTAGG-3’	450	95/240, 95/30 (35), 62.5/30, 72/30, 72/420
L0/H0	*cytb*	F: 5’-GGACAAATATCATTCTGAGG-3’ R: 5’-GGGTGTTCTACTGGTTGGCTTCC-3	589	94/300, 94/30 (35), 55/45, 72/60, 72/420
L0/H1	*cytb*	F: 5’-GGACAAATATCATTCTGAGG-3’ R: 5’-GGGTGGAATGGGATTTTGTC-3’	220	94/300, 94/30 (35), 65/45, 72/60, 72/420
DW2/DW4	*cytb*	F: 5’-TAATGCCTAGACGTATTCCTGATTATCCAG-3’ R: 5’- TGTTTGCTTGGGAGCTGTAATCATAATGTG-3’	800	94/240, 94/30 (35), 60/30, 68/50, 68/420
DW1/DW3	*cytb*	F: 5’-TCAACAATGACTTTATTTGG-3’ R: 5’-TGCTGTATCATACCCTAAAG-3’	1,000	94/60, 94/20 (40), 52/20, 68/30, 68/420

^*^Cycle conditions: temperature (°C), time (s), and number of cycles (in parentheses) for denaturation, annealing, and extension. Primers H2714/L2513; 16L1/H3056; L0/H0, and L0/H1 were used for host blood meal determination. Primers DW2/DW4 (outer) and DW1/DW6 (inner) were used for *P. odocoilei* detection. F, forward; R, reverse

Gel electrophoresis was performed to visualize the PCR products [39]. Amplicons from blood meals and *Plasmodium* infection assays were purified and sequenced using the Sanger method by Eurofins Genomics (Louisville, KY, USA). Nucleotide sequences were entered into the GenBank (National Center for Biotechnology Information) database using the Basic Local Alignment Search Tool (BLASTn). Only sequences with ≥ 95% identity matches determined by BLASTn were accepted for blood meal host and *Plasmodium* species identification [39].

### Statistics

A natural vector of deer malaria must take multiple (minimum of two) blood meals from WTD in its lifetime [19]. Therefore, putative vectors must specialize in feeding on this single host species to satisfy the incrimination criteria. We considered at least 75% of blood meals from WTD (three out of four) to constitute WTD host specialization.

The infection rate of each vector species was calculated by taking the number of samples positive for *P. odocoilei* and dividing them by the total females screened. Each positive pool was assumed to have only one female mosquito positive for *P. odocoilei* [40]. The “binom.test” function implemented in R Core Team (2024) was used to calculate 95% confidence (CI) intervals using the Clopper–Pearson “exact” interval [41].

## Results

### Trap type

**Fig. 1:**
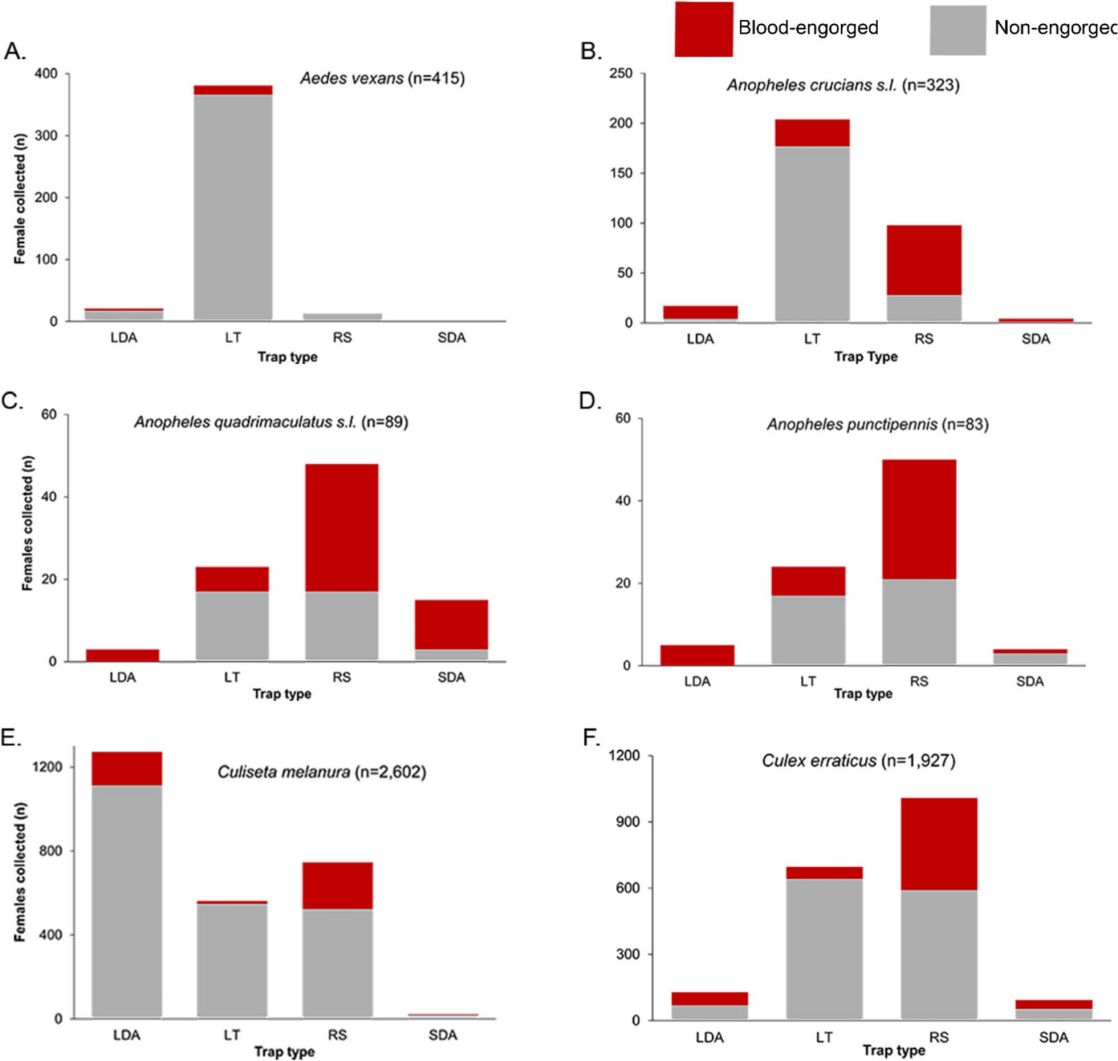
Relationship between trap type and physiological status for mosquito species at a deer farm in FL, USA. Traps included a large-diameter aspirator (LDA), Centers for Disease Control miniature light traps baited with carbon dioxide (LT), a pop-up resting shelter (RS), and a small diameter aspirator (SDA). **A** *Aedes vexans*; **B** *Anopheles crucians* s.l.; **C** *Anopheles quadrimaculatus* s.l.; **D** *Anopheles punctipennis* s.l.; **E** *Culiseta melanura*; **F** *Culex erraticus*

A total of 5957 female mosquitoes were collected using a combination of trapping methods, which included pop-up resting shelters, a large-diameter aspirator, a small-diameter aspirator, and a carbon-dioxide-baited miniature light trap (Fig. 1). Nine different genera were collected, including mosquitoes from the genera *Aedes* (8.8%, *n* = 523), *Anopheles* (8.5%, *n* = 509), *Culiseta* (43.8%, *n* = 2610), and *Culex* (36.8%, *n* = 2190). The most frequently collected species was *Culiseta melanura*, captured primarily by the large-diameter aspirator (48.9%, *n* = 1272), of which 12.5% (*n* = 159) were blood-engorged (Fig. 1). *Culex erraticus* constituted 32.6% (*n* = 1927) of total mosquitoes collected (Fig. 1), captured mainly by resting shelters (52.0%, *n* = 1008), with a large percentage being blood-engorged (41.3%, *n* = 416). The resting shelter also captured the largest percentage of *Anopheles punctipennis* (60.2%, *n* = 83) and *Anopheles quadrimaculatus* s.l. (53.0%, *n* = 89) (Fig. 1), with a large percentage being blood-engorged (59.6% and 64.6%, respectively). Most *An. crucians* s.l. were captured using light traps (62.2%, *n* = 323); however, the light traps captured a low percentage of blood-engorged *An. crucians* s.l. females (13.2%).

### Blood meal analysis

**Fig. 2:**
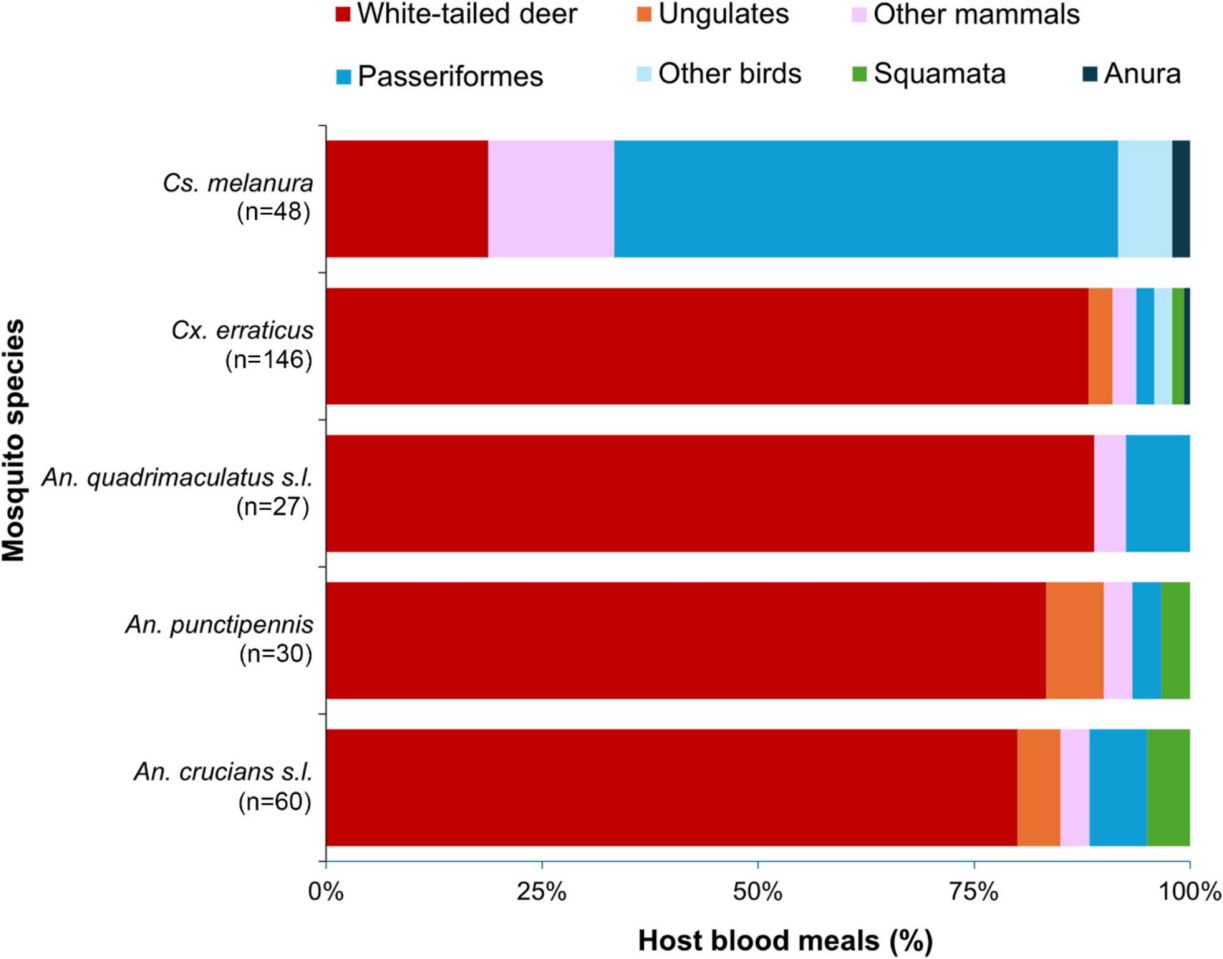
Host associations of selected mosquito species from a Florida deer farm. PCR-based blood meal analysis followed by Sanger sequencing was performed to determine the vertebrate host identity. Sample sizes provided in parentheses

A diverse assemblage of vertebrates was bitten by the mosquitoes sampled at the deer farm, varying substantially by mosquito species (Supplementary Table S1). A total of 632 blood meals were identified, derived from a wide range of species of the classes Aves (18 species), Mammalia (12 species), Reptilia (1 species), and Amphibia (1 species) (Fig. 1). In general, species of *Anopheles* took a large fraction (> 80%) of identified blood meals from mammals, particularly WTD (Fig. 2). *Anopheles quadrimaculatus* s.l. fed primarily on mammals (92.6%, 25/27), including a large percentage of total blood meals from WTD (88.9%; Fig. 2) and one blood meal from eastern cottontail rabbit (*Sylvilagus floridanus*) (3.7%). *Anopheles punctipennis* took 93.3% (28/30) of blood meals from mammals, including WTD (83.3%; Fig. 2), axis deer (*Axis axis*) (3.3%), and fallow deer (*Dama dama*) (3.3%). *Anopheles crucians* s.l. took 88.3% (53/59) of blood meals from mammals, with 85.0% of blood meals from Artiodactyla, specifically WTD (80.0%, Fig. 2), cows (*Bos taurus*) (3.3%), and blackbuck (*Antilope cervicapra*) (1.7%). Several species of songbird, particularly the northern cardinal (*Cardinalis cardinalis*), wood thrush (*Hylocichla mustelina*), red-eyed vireo (*Vireo olivaceus*), and common yellowthroat (*Geothlypis trichas*), were bitten occasionally (one blood meal or less per mosquito species) by *An. quadrimaculatus* s.l., *An. punctipennis*, and *An. crucians* s.l. *Anopheles punctipennis* (*n* = 1), and *Anopheles crucians* (*n* = 3) also bit the green anole lizard *Anolis carolinensis* (Fig. 2).

*Culex erraticus* took a large percentage of blood meals from mammals (93.2%, 136/146), mostly from Artiodactyla, including WTD (87.7%, Fig. 2), nilgai (*Boselaphus tragocamelus*) (1.4%), and fallow deer (1.4%). *Culex erraticus* fed upon diverse birds (4.7% of total blood meals), including single blood meals from a mallard (*Anas platyrhynchos*), green heron (*Butorides virescens*), veery (*Catharus fuscescens)*, yellow-rumped warbler (*Setophaga coronata)*, common yellowthroat, wild turkey (*Meleagris gallopavo)*, and barred owl (*Strix varia*). *Culex erraticus* also fed on an amphibian (southern toad [*Anaxyrus terrestris*]; 0.7%) and one reptile (green anole lizard; 1.4%).

*Culiseta melanura* mainly fed upon birds (64.6%, 31/48), which included red-eyed vireo (16.7%), wood thrush (14.6%), northern cardinal (6.3%), veery (4.2%), ruby-crowned kinglet (*Corthylio calendula*) (4.2%), yellow-rumped warbler (2.1%), yellow-billed cuckoo (*Coccyzus americanus*) (2.1%), white-eyed vireo (*Vireo griseus*) (2.1%), turkey vulture (*Cathartes aura*) (2.1%), tufted titmouse (*Baeolophus bicolor*) (2.1%), Swainson’s thrush (*Catharus ustulatus*) (2.1%), Carolina wren (*Thryothorus ludovicianus*) (2.1%), and barred owl (2.1%). A lower percentage of *Cs. melanura* blood meals were derived from mammals (33.3%, 16/48), including blood meals from WTD (18.8%), and single blood meals from five other mammal species (Fig. 2).

### *Plasmodium odocoilei* infection rate

*Anopheles quadrimaculatus* s.l., *An. punctipennis*, and *An. crucians* s.l. were the only species found infected with *P. odocoilei* at the deer farm (Table 2). The highest overall *P. odocoilei* infection rate was observed in *An. quadrimaculatus* s.l. (4.1%; Clopper–Pearson exact 95% CI 4.0–4.1%), followed by *An. punctipennis* (3.1%; Clopper–Pearson exact 95% CI 3.1–3.2%) and then *An. crucians* s.l. (0.47%; Clopper–Pearson exact 95% CI 0.46–0.48%). Two other species, *Cx. erraticus* and *Cs. melanura*, had large sample sizes screened for the parasite (*n* = 1457 and *n* = 1741, respectively), but *P. odocoilei* was not detected in those species. *Plasmodium odocoilei* was detected in *An. quadrimaculatus* s.l., at all sampling sites, with the highest infection rate observed at the barn pens (6.7%), followed by breeding pens (3.7%) and preserve (3.3%). In contrast, the highest infection rate for *An. punctipennis* was observed at the barn pens (6.7%), followed by breeding pens (3.2%), but was not detected at the preserve (0/15 screened). For *An. crucians* s.l., *P. odocoilei* was only detected at the preserve (1.1%), not at the breeding pens (*n* = 37 screened) or barn pens (*n* = 70 screened). *Plasmodium* sequences showed a high similarity to available *P. odocoilei* sequences in GenBank (range = 98.7–100%; mean = 99.9%), suggesting that the *Plasmodium* spp. detected were indeed *P. odocoilei*. For most of the mosquito pools (six of seven) that tested positive for *P. odocoilei*, a blood meal of a female that was included in the pool also tested positive for the parasite. Two *An. punctipennis* pools tested positive for *P. odocoilei*; however, only one of the corresponding blood meals was found to be positive. Two *An. quadrimaculatus* s.l. pools were positive for *P. odocoilei*, and positive blood meals were detected for both pools. For the single *An. crucians* s.l. pool that tested positive, a positive blood meal was also detected.

**Table 2:** Plasmodium odocoilei in mosquitoes from a deer farm in Gadsden County, Florida, USA (2022–2023)

Species	*P. odocoilei*	Breeding pens	Barn pens	Preserve	NA*	Total (CI)
*Anopheles crucians* s.l.	Females screened	37	70	95	11	213
	Positive	0	0	1	0	1
	Infection rate (%)	0	0	1.1	0	0.47 (0.46–0.48)
*Anopheles punctipennis*	Females screened	31	15	15	3	64
	Positive	1	1	0	0	2
	Infection rate (%)	3.2	6.7	0	0	3.1 (3.1–3.2)
*Anopheles quadrimaculatus* s.l.	Females screened	27	15	30	2	74
	Positive	1	1	1	0	3
	Infection rate (%)	3.7	6. 7	3.3	0	4.1 (4.0–4.1)
*Culex erraticus*	Females screened	274	802	328	53	1457
	Positive	0	0	0	0	0
	Infection rate (%)	0	0	0	0	0
*Culiseta melanura*	Females screened	399	286	972	84	1741
	Positive	0	0	0	0	0
	Infection rate (%)	0	0	0	0	0

^*^Sites marked as “NA” indicate that study locations were not recorded. Mosquitoes were sampled in three areas (breeding pens, barn pens, and preserve). Pools consisted of 25 females or fewer, screened by nested PCR-based assay targeting the *cytb* gene of Haemosporida followed by Sanger sequencing of amplicons

## Discussion

The objectives of this study were to determine which mosquito species specialize in feeding on WTD and to quantify the infection rate of *P. odocoilei* in female mosquitoes, thereby partially incriminating vector(s) of deer malaria. *Culiseta melanura*, *Cx. erraticus*, *An. punctipennis*, *An. quadrimaculatus* s.l., and *An. crucians* s.l. were present in areas inhabited by WTD at the farm. However, only *An. punctipennis*, *An. quadrimaculatus* s.l., and *An. crucians* s.l. specialized in feeding on WTD and were found to be infected with *P. odocoilei*. These results indicate that the putative vectors for *P. odocoilei* include *An. quadrimaculatus* s.l., *An. punctipennis*, and to a lesser extent, *An. crucians* s.l..

The possibility of *An. quadrimaculatus* s.l. being a vector for deer malaria is supported by its high specialization in feeding on WTD (88.9%) and relatively high *P. odocoilei* infection rate (4.1%). Other studies across North America have shown that *An. quadrimaculatus* takes a relatively high percentage of its blood meals (average of 60.0% across studies) from WTD (Table 3). Of the 12 available studies, 5 found that *An. quadrimaculatus* specialized on WTD, taking 75–100% of total blood meals from this single host species [11–14, 42–50]. These studies varied considerably in location, from zoos and urban residences to forests and swamps, potentially explaining some of the variation in the feeding patterns of *An. quadrimaculatus*. The detection of *Plasmodium* parasites in both the head–thorax segments and the corresponding blood meals of *An. quadrimaculatus* s.l. suggests that these females may have been infected with both sporozoites and gametocytes. Overall, evidence of *An. quadrimaculatus* s.l. specializing in feeding on WTD, its natural infection with *P. odocoilei*, and the potential presence of both gametocytes and sporozoites support its role as a potential vector of deer malaria.

**Table 3:** Summary of published blood meal analysis studies, with respect to feeding on white-tailed deer

Location	Setting	Cite	*An. crucians* s.l.	*An. punctipennis*	*An. quadrimaculatus* s.l.	*Cs. melanura*	*Cx. erraticus*
			(%) WTD (*n*)	(%) WTD (*n*)	(%) WTD (*n*)	(%) WTD (*n*)	(%) WTD (*n*)
Canada	Urban/forest	[14]	–	32 (21)	21 (14)	11 (9)	–
NY	Forest	[13]	–	96.3* (107)	97.7* (131)	–	–
CT	Urban	[12]	–	90.9* (11)	75.0* (8)	–	–
NJ	Rural	[43]	72.0 (50)	60.0 (20)	83.9* (415)	4.4 (68)	–
NJ	Forest	[13]	–	85.8* (127)	96.9* (288)	–	–
NC	Rural	[44]	25.0 (4)	50.0 (16)	7.7 (13)	0 (51)	0 (13)
NC	Urban	[45]	–	–	42.9 (550)	–	27.2 (169)
TN	Zoo	[46]	–	20.0 (5)	0 (7)	–	5.9 (17)
TN	Urban	[43]	–	–	14.2 (169)	–	10.0 (50)
TN	Urban	[47]	–	70.0 (10)	11.6 (249)	–	0.4 (454)
TN	Forest	[11]	–	29.0 (34)	40 (112)	0 (3)	60.0 (82)
AL	Rural	[50]	–	32.4 (34)	–	–	8.0 (25)
FL	Forest	[48]	22.7 (422)	–	55.0 (20)	–	–
FL	Swamp	[42]	28.6 (21)	–	–	–	19.7 (66)
FL	Swamp	[49]	54.5 (22)	–	44.1 (34)	1.7 (60)	19.2 (500)
FL	Rural	[51]	11.1 (9)	0 (5)	100* (1)	0 (57)	0 (24)
FL	Rural	[34]	–	–	–	0 (233)	–
FL	Rural	[56]				0.5 (213)	
Total			28.8 (528)	71.3 (390)	60.0 (2011)	1.0 (694)	15.3 (1400)

Asterisks denote > 75% of feeding on white-tailed deer (WTD)

*Anopheles punctipennis* is also a likely vector of *P. odocoilei*, owing to specialization in feeding on WTD in this study (83.3%) and natural infection (3.1%) with *P. odocoilei* in field-caught samples. Other studies showed that *An. punctipennis* also takes a relatively high percentage of blood meals from WTD in nature (average of 71.3%). Of 12 available blood meal analysis studies, 3 reported that *An. punctipennis* is specialized in feeding on WTD, taking 75–100% of total blood meals from this single host species [11–14, 43–51]. The detection *P. odocoilei* in the head–thorax and the corresponding blood meals of *An. punctipennis* suggest that sporozoites and gametocytes could be present. The absence of *P. odocoilei* in blood meals from females whose bodies were pooled suggests that some females harbored sporozoite stage, potentially indicating infectious vectors. The likelihood of *An. punctipennis* being a vector for *P. odocoilei* is also supported by a study from Washington, DC, [9] showing a similar infection rate of *P. odocoilei* in *An. punctipennis* (5.0%) as in our study (3.1%).

*Anopheles crucians* s.l. may serve as a vector for *P. odocoilei*. However, owing to its relatively low *P. odocoilei* infection rate in our study (0.47%), this species likely does not play a major role in transmission. Although *An. crucians* took an average of 28.8% of its total blood meals from WTD in other studies (Table 3), this species was not observed to specialize in feeding on WTD in the seven available studies [43–45, 49, 50]. The low infection rate of *P. odocoilei*, low percentage of blood meals from WTD, and the *Plasmodium*-infected blood meal suggest that *An. crucians* s.l. plays a minor role in the transmission of deer malaria.

Other common species of mosquitoes observed in our study, particularly *Cx. erraticus* and *Cs. melanura*, are unlikely vectors of *P. odocoilei*. Although *Cx. erraticus* showed a high specialization in feeding on WTD in our study (87.7%), it did not specialize in feeding on WTD in nine other studies, where an average of 15.3% of its blood meals came from WTD [11, 42–47, 49–51]. In contrast, *Cs. melanura* mainly fed on songbirds on the deer farm in our study. Other studies found that *Cs. melanura* does not specialize in feeding on WTD either (1.0% on average) [11, 14, 34, 42–44, 49, 51]. Neither *Cx. erraticus* nor *Cs. melanura* were found infected with *P. odocoilei*, despite over 1000 females of each species being screened for the parasite. Given the low percentage of blood meals from WTD and lack of infection, both species are not likely vectors of deer malaria.

This study was conducted at a single deer farm in Gadsden County, which may limit the application of findings to other regions. In addition, females of the *An. quadrimaculatus* complex and *An. crucians* complex are difficult to reliably identify morphologically, so the dominant member within each group is unclear. Future research could use DNA barcoding to determine species composition at the deer farm and assess whether host use or *P. odocoilei* infection rates vary among members of each group.

Despite our efforts to separate thoraxes and abdomens when processing samples, PCR-based detection of *P. odocoilei* is unable to specifically determine the stage of the parasite. Detection of *P. odocoilei* in the thorax could be attributed to the amplification of infected blood meal residue, although this is unlikely owing to degradation by mosquito digestive enzymes [52, 53]. Alternatively, it is possible that PCR amplification represented underdeveloped oocysts or nonviable sporozoites present in the hemocoel [53, 54]. Future studies could perform dissections and microscopy, as well as develop molecular markers to target parasite stage-specific proteins, to clarify whether or not PCR positives represent infectious mosquitoes [52, 53]. Despite these limitations in the collection and blood meal analysis, the study successfully identified mosquitoes specializing in feeding on WTD at the study site and detected the *P. odocoilei* in wild-collected adult females of three mosquito species.

## Conclusions

This study is important for partially incriminating the vectors of *P. odocoilei* and understanding the transmission of deer malaria. *Anopheles quadrimaculatus* s.l. and *An. punctipennis* better met criteria for vector incrimination than *An. crucians* s.l.. Their relative importance as vectors of *P. odocoilei* likely varies with geographic region and habitat. *Anopheles punctipennis* is more common in temperate woodlands, while *Anopheles quadrimaculatus* s.l. is associated with forested wetlands [29]. Future studies should evaluate the vector competence of *An. quadrimaculatus* s.l.., *An. punctipennis*, and *An. crucians* s.l. for transmitting *P. odocoilei* in laboratory settings. If vector competence is confirmed, IPM strategies that focus on the vector species can be developed to decrease the risk of infection in WTD, particularly on deer farms [55].

## Supplementary information

Supplementary material 1.

## Data Availability

Data are provided within the manuscript or supplementary information files.

## Abbreviations

WTD: White-tailed deer
PCR: Polymerase chain reaction
EHDV: Epizootic hemorrhagic disease virus
CDC: Centers for Disease Control and Prevention
*cytb*: *Cytochrome* *b* gene
