# Supplementary material for: Incriminating vectors of deer malaria (Plasmodium odocoilei) at a Florida deer farm
Source: Parasit Vectors. 2025 Jul 28;18:308. doi: 10.1186/s13071-025-06942-5 (PMC12305987; doi:10.1186/s13071-025-06942-5)
Supplement: Supplementary file 1 — Supplementary material 1. [file 13071_2025_6942_MOESM1_ESM.docx]

Table S1. Host species of mosquitoes sampled from a commercial deer farm, Gadsden County, Florida USA.

| Mosquito species | Date | County | Area | Host Class | Host species |
| --- | --- | --- | --- | --- | --- |
| *Anopheles quadrimaculatus* | 6/25/24 | Gadsden | Preserve | - | - |
| *Culex nigripalpus* | 5/16/23 | Gadsden | Breeding pens | Mammalia | *Odocoileus virginianus* |
| *Anopheles quadrimaculatus* | 7/4/23 | Gadsden | Barn pens | Mammalia | *Odocoileus virginianus* |
| *Anopheles quadrimaculatus* | 6/25/24 | Gadsden | Preserve | - | - |
| *Culex erraticus* | 5/18/23 | Gadsden | Barn pens | Mammalia | *Odocoileus virginianus* |
| *Anopheles quadrimaculatus* | 7/4/23 | Gadsden | Barn pens | Mammalia | *Odocoileus virginianus* |
| *Culex erraticus* | 6/25/24 | Gadsden | Preserve | - | - |
| *Culex erraticus* | 7/4/23 | Gadsden | Barn pens | Mammalia | *Odocoileus virginianus* |
| *Anopheles punctipennis* | 5/17/23 | Gadsden | Breeding pens | Mammalia | *Odocoileus virginianus* |
| *Anopheles crucians* | 6/25/24 | Gadsden | Preserve | - | - |
| *Culex erraticus* | 5/18/23 | Gadsden | Breeding pens | Mammalia | *Odocoileus virginianus* |
| *Culex erraticus* | 7/4/23 | Gadsden | Barn pens | Mammalia | *Odocoileus virginianus* |
| *Anopheles crucians* | 6/25/24 | Gadsden | Preserve | - | - |
| *Culex erraticus* | 7/4/23 | Gadsden | Barn pens | Mammalia | *Odocoileus virginianus* |
| *Culex erraticus* | 5/17/23 | Gadsden | Barn pens | Mammalia | *Odocoileus virginianus* |
| *Culex erraticus* | 5/20/23 | Gadsden | Barn pens | - | - |
| *Culex erraticus* | 6/25/24 | Gadsden | Preserve | - | - |
| *Culex erraticus* | 7/4/23 | Gadsden | Barn pens | Mammalia | *Odocoileus virginianus* |
| *Culex erraticus* | 6/25/24 | Gadsden | Preserve | - | - |
| *Culex erraticus* | 7/4/23 | Gadsden | Barn pens | Mammalia | *Odocoileus virginianus* |
| *Anopheles crucians* | 5/17/24 | Gadsden | Preserve | Mammalia | *Odocoileus virginianus* |
| *Culiseta melanura* | 5/18/23 | Gadsden | Barn pens | Mammalia | *Rattus norvegicus* |
| *Culex erraticus* | 6/25/24 | Gadsden | Preserve | - | - |
| *Culex erraticus* | 7/4/23 | Gadsden | Barn pens | Mammalia | *Odocoileus virginianus* |
| *Culiseta melanura* | 5/18/23 | Gadsden | Breeding pens | Mammalia | *Odocoileus virginianus* |
| *Culex erraticus* | 7/4/23 | Gadsden | Barn pens | Mammalia | *Odocoileus virginianus* |
| *Culex erraticus* | 5/18/23 | Gadsden | Barn pens | Mammalia | *Odocoileus virginianus* |
| *Culex erraticus* | 6/25/24 | Gadsden | Preserve | - | - |
| *Culex erraticus* | 7/4/23 | Gadsden | Barn pens | Mammalia | *Odocoileus virginianus* |
| *Culex erraticus* | 6/25/24 | Gadsden | Barn pens | - | - |
| *Culex erraticus* | 7/4/23 | Gadsden | Barn pens | Mammalia | *Odocoileus virginianus* |
| *Anopheles crucians* | 5/14/24 | Gadsden | Breeding pens | Mammalia | *Sylvilagus floridanus* |
| *Culex erraticus* | 7/4/23 | Gadsden | Barn pens | Mammalia | *Odocoileus virginianus* |
| *Culex erraticus* | 7/4/23 | Gadsden | Barn pens | Aves | *Strix varia* |
| *Anopheles crucians* | 6/25/24 | Gadsden | Breeding pens | Mammalia | *Odocoileus virginianus* |
| *Anopheles crucians* | 6/25/24 | Gadsden | Breeding pens | Mammalia | *Odocoileus virginianus* |
| *Culex erraticus* | 6/25/24 | Gadsden | Breeding pens | - | - |
| *Culex erraticus* | 7/4/23 | Gadsden | Preserve | Aves | *Geothlypis trichas* |
| *Anopheles crucians* | 5/15/24 | Gadsden | Preserve | Aves | *Geothlypis trichas* |
| *Culex erraticus* | 6/25/24 | Gadsden | Barn pens | - | - |
| *Culex erraticus* | 7/4/23 | Gadsden | Preserve | Mammalia | *Odocoileus virginianus* |
| *Anopheles crucians* | 6/25/24 | Gadsden | Breeding pens | Mammalia | *Odocoileus virginianus* |
| *Anopheles quadrimaculatus* | 6/25/24 | Gadsden | Barn pens | - | - |
| *Culex erraticus* | 7/7/23 | Gadsden | Barn pens | Aves | *Anas platyrhynchos* |
| *Anopheles crucians* | 6/25/24 | Gadsden | Breeding pens | - | - |
| *Culex erraticus* | 7/4/23 | Gadsden | Barn pens | Mammalia | *Odocoileus virginianus* |
| *Culex erraticus* | 6/25/24 | Gadsden | Barn pens | - | - |
| *Culex erraticus* | 7/6/23 | Gadsden | Preserve | Mammalia | *Odocoileus virginianus* |
| *Anopheles crucians* | 6/25/24 | Gadsden | Breeding pens | - | - |
| *Culiseta melanura* | 7/6/23 | Gadsden | Preserve | Aves | *Vireo olivaceus* |
| *Culex erraticus* | 6/25/24 | Gadsden | Breeding pens | - | - |
| *Culex erraticus* | 7/7/23 | Gadsden | Breeding pens | Mammalia | *Odocoileus virginianus* |
| *Anopheles crucians* | 6/25/24 | Gadsden | Breeding pens | - | - |
| *Anopheles crucians* | 5/15/24 | Gadsden | Breeding pens | Mammalia | *Odocoileus virginianus* |
| *Culex erraticus* | 7/7/23 | Gadsden | Barn pens | Mammalia | *Odocoileus virginianus* |
| *Culex erraticus* | 6/25/24 | Gadsden | Breeding pens | - | - |
| *Culex erraticus* | 7/7/23 | Gadsden | Barn pens | Mammalia | *Odocoileus virginianus* |
| *Culex erraticus* | 6/25/24 | Gadsden | Barn pens | - | - |
| *Culex erraticus* | 6/25/24 | Gadsden | Barn pens | - | - |
| *Culex erraticus* | 7/7/23 | Gadsden | Barn pens | Mammalia | *Odocoileus virginianus* |
| *Culex erraticus* | 6/25/24 | Gadsden | Barn pens | - | - |
| *Anopheles crucians* | 5/16/24 | Gadsden | Breeding pens | Mammalia | *Odocoileus virginianus* |
| *Culex erraticus* | 7/7/23 | Gadsden | Barn pens | Mammalia | *Odocoileus virginianus* |
| *Culex erraticus* | 6/25/24 | Gadsden | Barn pens | - | - |
| *Anopheles crucians* | 5/16/24 | Gadsden | Breeding pens | Mammalia | *Odocoileus virginianus* |
| *Culex erraticus* | 7/7/23 | Gadsden | Barn pens | Mammalia | *Odocoileus virginianus* |
| *Culex erraticus* | 7/7/23 | Gadsden | Barn pens | - | - |
| *Culiseta melanura* | 7/6/23 | Gadsden | Preserve | Aves | *Vireo olivaceus* |
| *Culex erraticus* | 6/25/24 | Gadsden | Barn pens | - | - |
| *Culex erraticus* | 7/7/23 | Gadsden | Barn pens | Mammalia | *Odocoileus virginianus* |
| *Culiseta melanura* | 5/15/24 | Gadsden | Breeding pens | Mammalia | *Odocoileus virginianus* |
| *Anopheles quadrimaculatus* | 6/25/24 | Gadsden | Barn pens | - | - |
| *Culex erraticus* | 7/7/23 | Gadsden | Barn pens | Mammalia | *Odocoileus virginianus* |
| *Anopheles quadrimaculatus* | 6/25/24 | Gadsden | Barn pens | Mammalia | *Odocoileus virginianus* |
| *Anopheles punctipennis* | 6/25/24 | Gadsden | Barn pens | - | - |
| *Culex erraticus* | 7/7/23 | Gadsden | Barn pens | Mammalia | *Odocoileus virginianus* |
| *Anopheles crucians* | 5/15/24 | Gadsden | Breeding pens | Mammalia | *Odocoileus virginianus* |
| *Culex erraticus* | 7/7/23 | Gadsden | Barn pens | - | - |
| *Culex erraticus* | 6/25/24 | Gadsden | Barn pens | - | - |
| *Anopheles crucians* | 5/19/24 | Gadsden | Breeding pens | Mammalia | *Odocoileus virginianus* |
| *Anopheles quadrimaculatus* | 6/29/24 | Gadsden | Barn pens | - | - |
| *Culex erraticus* | 7/8/23 | Gadsden | Barn pens | Mammalia | *Odocoileus virginianus* |
| *Anopheles crucians* | 5/16/24 | Gadsden | Breeding pens | Mammalia | *Odocoileus virginianus* |
| *Anopheles quadrimaculatus* | 6/25/24 | Gadsden | Barn pens | Mammalia | *Odocoileus virginianus* |
| *Culex erraticus* | 7/8/23 | Gadsden | Barn pens | Mammalia | *Odocoileus virginianus* |
| *Anopheles punctipennis* | 6/25/24 | Gadsden | Barn pens | Aves | *Cardinalis cardinalis* |
| *Culex erraticus* | 7/8/23 | Gadsden | Barn pens | - | - |
| *Culex erraticus* | 7/8/23 | Gadsden | Barn pens | Mammalia | *Odocoileus virginianus* |
| *Culex erraticus* | 6/25/24 | Gadsden | Barn pens | - | - |
| *Culex erraticus* | 6/25/24 | Gadsden | Barn pens | - | - |
| *Anopheles crucians* | 5/14/24 | Gadsden | Breeding pens | Mammalia | *Odocoileus virginianus* |
| *Culex erraticus* | 7/8/23 | Gadsden | Barn pens | Mammalia | *Odocoileus virginianus* |
| *Anopheles crucians* | 10/13/22 | Gadsden | Breeding pens | Aves | *Hylocichla mustelina* |
| *Culex erraticus* | 7/8/23 | Gadsden | Barn pens | Mammalia | *Odocoileus virginianus* |
| *Anopheles quadrimaculatus* | 6/25/24 | Gadsden | Breeding pens | Mammalia | *Odocoileus virginianus* |
| *Anopheles punctipennis* | 6/25/24 | Gadsden | Breeding pens | - | - |
| *Culex erraticus* | 7/7/23 | Gadsden | Barn pens | Mammalia | *Odocoileus virginianus* |
| *Anopheles quadrimaculatus* | 6/25/24 | Gadsden | Barn pens | Mammalia | *Odocoileus virginianus* |
| *Culex erraticus* | 7/7/23 | Gadsden | Barn pens | - | - |
| *Anopheles crucians* | 6/25/24 | Gadsden | Breeding pens | - | - |
| *Anopheles quadrimaculatus* | 6/25/24 | Gadsden | Breeding pens | - | - |
| *Culex erraticus* | 7/7/23 | Gadsden | Barn pens | Amphibia | *Anaxyrus terrestris* |
| *Culex erraticus* | 7/7/23 | Gadsden | Barn pens | Mammalia | *Odocoileus virginianus* |
| *Culiseta melanura* | 6/25/24 | Gadsden | Breeding pens | - | - |
| *Anopheles punctipennis* | 6/26/24 | Gadsden | Preserve | Mammalia | *Odocoileus virginianus* |
| *Culex erraticus* | 7/7/23 | Gadsden | Barn pens | Mammalia | *Odocoileus virginianus* |
| *Anopheles punctipennis* | 6/26/24 | Gadsden | Preserve | - | - |
| *Culex erraticus* | 7/7/23 | Gadsden | Barn pens | Mammalia | *Dama dama* |
| *Anopheles quadrimaculatus* | 6/26/24 | Gadsden | Preserve | Mammalia | *Odocoileus virginianus* |
| *Culex erraticus* | 7/7/23 | Gadsden | Barn pens | - | - |
| *Culex erraticus* | 6/26/24 | Gadsden | Preserve | - | - |
| *Anopheles punctipennis* | 6/26/24 | Gadsden | Preserve | - | - |
| *Culex erraticus* | 7/7/23 | Gadsden | Barn pens | Mammalia | *Odocoileus virginianus* |
| *Culex erraticus* | 7/7/23 | Gadsden | Barn pens | Mammalia | *Odocoileus virginianus* |
| *Anopheles quadrimaculatus* | 6/26/24 | Gadsden | Preserve | - | - |
| *Anopheles quadrimaculatus* | 7/7/23 | Gadsden | Preserve | Mammalia | *Odocoileus virginianus* |
| *Culex erraticus* | 7/8/23 | Gadsden | Barn pens | - | - |
| *Anopheles quadrimaculatus* | 6/26/24 | Gadsden | Preserve | Aves | *Cardinalis cardinalis* |
| *Culex erraticus* | 7/8/23 | Gadsden | Barn pens | - | - |
| *Culex erraticus* | 6/26/24 | Gadsden | Preserve | - | - |
| *Anopheles quadrimaculatus* | 6/26/24 | Gadsden | Preserve | Mammalia | *Odocoileus virginianus* |
| *Anopheles crucians* | 8/17/23 | Gadsden | Breeding pens | - | - |
| *Culex erraticus* | 7/8/23 | Gadsden | Barn pens | - | - |
| *Culex erraticus* | 6/26/24 | Gadsden | Preserve | - | - |
| *Anopheles quadrimaculatus* | 6/25/24 | Gadsden | Breeding pens | - | - |
| *Culiseta melanura* | 6/26/24 | Gadsden | Breeding pens | - | - |
| *Culex erraticus* | 7/5/23 | Gadsden | Barn pens | Mammalia | *Odocoileus virginianus* |
| *Culex erraticus* | 6/27/24 | Gadsden | Preserve | - | - |
| *Culex erraticus* | 7/5/23 | Gadsden | Barn pens | Mammalia | *Odocoileus virginianus* |
| *Culex erraticus* | 7/5/23 | Gadsden | Barn pens | Mammalia | *Odocoileus virginianus* |
| *Culex erraticus* | 6/27/24 | Gadsden | Preserve | - | - |
| *Culex erraticus* | 7/5/23 | Gadsden | Barn pens | Mammalia | *Odocoileus virginianus* |
| *Culex erraticus* | 7/5/23 | Gadsden | Barn pens | Mammalia | *Odocoileus virginianus* |
| *Culex erraticus* | 6/27/24 | Gadsden | Preserve | - | - |
| *Culex erraticus* | 7/5/23 | Gadsden | Barn pens | Mammalia | *Odocoileus virginianus* |
| *Anopheles punctipennis* | 6/27/24 | Gadsden | Breeding pens | - | - |
| *Culex erraticus* | 7/5/23 | Gadsden | Barn pens | Mammalia | *Odocoileus virginianus* |
| *Culex erraticus* | 7/5/23 | Gadsden | Barn pens | Mammalia | *Odocoileus virginianus* |
| *Culex erraticus* | 7/5/23 | Gadsden | Barn pens | Mammalia | *Odocoileus virginianus* |
| *Culex erraticus* | 7/6/23 | Gadsden | Barn pens | Mammalia | *Odocoileus virginianus* |
| *Culex erraticus* | 6/27/24 | Gadsden | Breeding pens | - | - |
| *Culex erraticus* | 7/5/23 | Gadsden | Barn pens | Mammalia | *Odocoileus virginianus* |
| *Culex erraticus* | 7/5/23 | Gadsden | Barn pens | - | - |
| *Culex erraticus* | 6/27/24 | Gadsden | Barn pens | - | - |
| *Anopheles quadrimaculatus* | 6/27/24 | Gadsden | Breeding pens | Mammalia | *Odocoileus virginianus* |
| *Culex erraticus* | 6/27/24 | Gadsden | Barn pens | - | - |
| *Culex erraticus* | 7/5/23 | Gadsden | Barn pens | Mammalia | *Odocoileus virginianus* |
| *Anopheles quadrimaculatus* | 6/27/24 | Gadsden | Breeding pens | Mammalia | *Odocoileus virginianus* |
| *Anopheles quadrimaculatus* | 6/27/24 | Gadsden | Breeding pens | - | - |
| *Culex erraticus* | 7/5/23 | Gadsden | Barn pens | Mammalia | *Boselaphus tragocamelus* |
| *Anopheles quadrimaculatus* | 6/27/24 | Gadsden | Breeding pens | Mammalia | *Odocoileus virginianus* |
| *Culex erraticus* | 6/27/24 | Gadsden | Breeding pens | - | - |
| *Anopheles punctipennis* | 7/5/23 | Gadsden | Barn pens | Mammalia | *Odocoileus virginianus* |
| *Culex erraticus* | 6/27/24 | Gadsden | Barn pens | - | - |
| *Anopheles quadrimaculatus* | 7/5/23 | Gadsden | Barn pens | Mammalia | *Odocoileus virginianus* |
| *Culex erraticus* | 7/8/23 | Gadsden | Barn pens | - | - |
| *Culiseta melanura* | 10/11/22 | Gadsden | Preserve | - | - |
| *Culex erraticus* | 7/5/23 | Gadsden | Barn pens | Mammalia | *Odocoileus virginianus* |
| *Culex erraticus* | 7/8/23 | Gadsden | Barn pens | - | - |
| *Anopheles spp. undetermined* | 10/12/22 | Gadsden | Preserve | - | - |
| *Culex erraticus* | 7/5/23 | Gadsden | Barn pens | Mammalia | *Odocoileus virginianus* |
| *Culex erraticus* | 6/27/24 | Gadsden | Barn pens | - | - |
| *Culex erraticus* | 7/5/23 | Gadsden | Barn pens | Mammalia | *Odocoileus virginianus* |
| *Culiseta melanura* | 10/12/22 | Gadsden | Preserve | Aves | *Catharus fuscescens* |
| *Culex erraticus* | 7/8/23 | Gadsden | Barn pens | Aves | *Butorides virescens* |
| *Culiseta melanura* | 10/12/22 | BT | NA | - | - |
| *Culiseta melanura* | 10/12/22 | BT | NA | - | - |
| *Culex erraticus* | 6/27/24 | Gadsden | Barn pens | - | - |
| *Culex erraticus* | 6/27/24 | Gadsden | Barn pens | - | - |
| *Anopheles punctipennis* | 7/5/23 | Gadsden | Barn pens | Mammalia | *Odocoileus virginianus* |
| *Anopheles quadrimaculatus* | 6/26/24 | Gadsden | Barn pens | - | - |
| *Culiseta melanura* | 10/10/22 | LO | NA | Mammalia | *Odocoileus virginianus* |
| *Culex erraticus* | 7/5/23 | Gadsden | Barn pens | Mammalia | *Odocoileus virginianus* |
| *Culex erraticus* | 10/12/22 | BT | NA | Mammalia | *Odocoileus virginianus* |
| *Culex erraticus* | 7/5/23 | Gadsden | Barn pens | Mammalia | *Odocoileus virginianus* |
| *Anopheles crucians* | 10/11/22 | Gadsden | Preserve | - | - |
| *Culex erraticus* | 6/26/24 | Gadsden | Barn pens | - | - |
| *Culex erraticus* | 7/5/23 | Gadsden | Barn pens | Mammalia | *Odocoileus virginianus* |
| *Culiseta melanura* | 10/12/22 | Gadsden | Preserve | - | - |
| *Culiseta melanura* | 6/27/24 | Gadsden | Preserve | - | - |
| *Culiseta melanura* | 10/12/22 | Gadsden | Preserve | Aves | *Catharus ustulatus* |
| *Culex erraticus* | 7/5/23 | Gadsden | Breeding pens | Mammalia | *Odocoileus virginianus* |
| *Culiseta melanura* | 10/12/22 | Gadsden | Preserve | - | - |
| *Culex erraticus* | 6/27/24 | Gadsden | Preserve | - | - |
| *Culex erraticus* | 7/8/23 | Gadsden | Barn pens | Mammalia | *Odocoileus virginianus* |
| *Anopheles crucians* | 10/12/22 | BT | NA | Mammalia | *Odocoileus virginianus* |
| *Culex erraticus* | 7/8/23 | Gadsden | Barn pens | Mammalia | *Odocoileus virginianus* |
| *Culex erraticus* | 7/8/23 | Gadsden | Barn pens | Mammalia | *Dama dama* |
| *Culex erraticus* | 10/12/22 | BT | NA | Mammalia | *Odocoileus virginianus* |
| *Culiseta melanura* | 10/13/22 | Gadsden | Preserve | Aves | *Regulus calendula* |
| *Culex erraticus* | 7/8/23 | Gadsden | Barn pens | Mammalia | *Odocoileus virginianus* |
| *Culex erraticus* | 10/13/22 | Gadsden | Preserve | Mammalia | *Sylvilagus floridanus* |
| *Culex erraticus* | 7/8/23 | Gadsden | Barn pens | Mammalia | *Odocoileus virginianus* |
| *Culiseta melanura* | 10/13/22 | Gadsden | Preserve | Aves | *Cardinalis cardinalis* |
| *Culex erraticus* | 7/8/23 | Gadsden | Barn pens | Mammalia | *Odocoileus virginianus* |
| *Culex erraticus* | 7/4/23 | Gadsden | Breeding pens | - | - |
| *Culiseta melanura* | 10/13/22 | Gadsden | Preserve | Aves | *Regulus calendula* |
| *Culex erraticus* | 7/4/23 | Gadsden | Breeding pens | Mammalia | *Odocoileus virginianus* |
| *Culiseta melanura* | 10/13/22 | Gadsden | Preserve | Aves | *Hylocichla mustelina* |
| *Culex erraticus* | 7/4/23 | Gadsden | Breeding pens | Mammalia | *Odocoileus virginianus* |
| *Culiseta melanura* | 10/13/22 | Gadsden | Preserve | Aves | *Vireo olivaceus* |
| *Culex erraticus* | 7/4/23 | Gadsden | Breeding pens | - | - |
| *Culiseta melanura* | 10/13/22 | Gadsden | Preserve | Aves | *Vireo olivaceus* |
| *Culiseta melanura* | 10/13/22 | Gadsden | Preserve | Aves | *Vireo olivaceus* |
| *Culex erraticus* | 7/4/23 | Gadsden | Breeding pens | Mammalia | *Odocoileus virginianus* |
| *Culex erraticus* | 6/28/24 | Gadsden | Barn pens | - | - |
| *Culiseta melanura* | 10/13/22 | Gadsden | Preserve | Aves | *Vireo olivaceus* |
| *Culiseta melanura* | 8/12/23 | Gadsden | Barn pens | Aves | *Vireo olivaceus* |
| *Culex erraticus* | 6/28/24 | Gadsden | Barn pens | - | - |
| *Culex erraticus* | 9/26/23 | Gadsden | Preserve | Mammalia | *Odocoileus virginianus* |
| *Culiseta melanura* | 10/13/22 | Gadsden | Preserve | Aves | *Dendroica coronata* |
| *Culex erraticus* | 8/16/23 | Gadsden | Barn pens | Aves | *Dendroica coronata* |
| *Culex erraticus* | 10/11/22 | Gadsden | Preserve | - | - |
| *Culex erraticus* | 6/28/24 | Gadsden | Barn pens | - | - |
| *Culex erraticus* | 9/26/23 | Gadsden | Preserve | Mammalia | *Odocoileus virginianus* |
| *Culex erraticus* | 8/16/23 | Gadsden | Barn pens | Mammalia | *Odocoileus virginianus* |
| *Culex erraticus* | 10/13/22 | Gadsden | Preserve | - | - |
| *Culex erraticus* | 6/28/24 | Gadsden | Barn pens | - | - |
| *Culex erraticus* | 8/16/23 | Gadsden | Barn pens | Mammalia | *Odocoileus virginianus* |
| *Culiseta melanura* | 10/13/22 | Gadsden | Preserve | - | - |
| *Culex erraticus* | 6/28/24 | Gadsden | Barn pens | - | - |
| *Culex erraticus* | 8/16/23 | Gadsden | Barn pens | Mammalia | *Odocoileus virginianus* |
| *Culex erraticus* | 8/16/23 | Gadsden | Barn pens | - | - |
| *Culex erraticus* | 6/28/24 | Gadsden | Barn pens | - | - |
| *Culex erraticus* | 6/28/24 | Gadsden | Barn pens | - | - |
| *Culex erraticus* | 8/16/23 | Gadsden | Barn pens | Mammalia | *Odocoileus virginianus* |
| *Culex erraticus* | 6/28/24 | Gadsden | Barn pens | - | - |
| *Anopheles crucians* | 6/25/24 | Gadsden | Preserve | Mammalia | *Odocoileus virginianus* |
| *Culex erraticus* | 8/16/23 | Gadsden | Barn pens | Mammalia | *Odocoileus virginianus* |
| *Culex erraticus* | 8/16/23 | Gadsden | Barn pens | - | - |
| *Culex erraticus* | 6/28/24 | Gadsden | Barn pens | - | - |
| *Culex erraticus* | 8/16/23 | Gadsden | Barn pens | - | - |
| *Culex erraticus* | 6/28/24 | Gadsden | Barn pens | - | - |
| *Culex erraticus* | 6/28/24 | Gadsden | Preserve | - | - |
| *Culex erraticus* | 8/16/23 | Gadsden | Preserve | Mammalia | *Odocoileus virginianus* |
| *Anopheles crucians* | 6/28/24 | Gadsden | Preserve | - | - |
| *Anopheles crucians* | 6/28/24 | Gadsden | Preserve | Mammalia | *Odocoileus virginianus* |
| *Culex erraticus* | 8/16/23 | Gadsden | Breeding pens | Mammalia | *Odocoileus virginianus* |
| *Culiseta melanura* | 8/16/23 | Gadsden | Breeding pens | - | - |
| *Culiseta melanura* | 8/16/23 | Gadsden | Breeding pens | - | - |
| *Anopheles crucians* | 8/12/23 | Gadsden | Preserve | Mammalia | *Odocoileus virginianus* |
| *Culex erraticus* | 8/12/23 | Gadsden | Barn pens | Mammalia | *Odocoileus virginianus* |
| *Culex erraticus* | 8/12/23 | Gadsden | NA | Mammalia | *Odocoileus virginianus* |
| *Culex erraticus* | 8/12/23 | Gadsden | NA | Mammalia | *Odocoileus virginianus* |
| *Culex spp. undetermined* | 8/12/23 | Gadsden | Barn pens | Mammalia | *Odocoileus virginianus* |
| *Culex erraticus* | 8/12/23 | Gadsden | Barn pens | Mammalia | *Odocoileus virginianus* |
| *Culex erraticus* | 8/12/23 | Gadsden | Barn pens | Mammalia | *Odocoileus virginianus* |
| *Anopheles quadrimaculatus* | 6/26/24 | Gadsden | Breeding pens | Mammalia | *Odocoileus virginianus* |
| *Culex erraticus* | 8/12/23 | Gadsden | Barn pens | Mammalia | *Odocoileus virginianus* |
| *Culex erraticus* | 8/12/23 | Gadsden | Barn pens | - | - |
| *Culex erraticus* | 8/12/23 | Gadsden | Barn pens | - | - |
| *Culex erraticus* | 8/12/23 | Gadsden | Barn pens | Mammalia | *Odocoileus virginianus* |
| *Culex erraticus* | 8/12/23 | Gadsden | Barn pens | - | - |
| *Culex erraticus* | 8/12/23 | Gadsden | Barn pens | Mammalia | *Odocoileus virginianus* |
| *Culiseta melanura* | 10/13/22 | Gadsden | Preserve | Aves | *Hylocichla mustelina* |
| *Culex erraticus* | 8/12/23 | Gadsden | Barn pens | Mammalia | *Odocoileus virginianus* |
| *Culiseta melanura* | 10/13/22 | Gadsden | Preserve | - | - |
| *Culex erraticus* | 8/12/23 | Gadsden | Barn pens | - | - |
| *Culex erraticus* | 8/12/23 | Gadsden | Barn pens | - | - |
| *Culiseta melanura* | 10/13/22 | Gadsden | Preserve | - | - |
| *Anopheles quadrimaculatus* | 6/25/24 | Gadsden | Barn pens | Mammalia | *Odocoileus virginianus* |
| *Culex erraticus* | 8/12/23 | Gadsden | Barn pens | - | - |
| *Culiseta melanura* | 10/13/22 | Gadsden | Preserve | - | - |
| *Culiseta melanura* | 10/11/22 | Gadsden | Preserve | - | - |
| *Culex erraticus* | 8/12/23 | Gadsden | Barn pens | - | - |
| *Culex erraticus* | 10/13/22 | Gadsden | Preserve | Mammalia | *Boselaphus tragocamelus* |
| *Culex erraticus* | 7/8/23 | Gadsden | Preserve | Mammalia | *Odocoileus virginianus* |
| *Anopheles punctipennis* | 7/8/23 | Gadsden | Breeding pens | - | - |
| *Culiseta melanura* | 10/13/22 | Gadsden | Preserve | - | - |
| *Culex erraticus* | 7/8/23 | Gadsden | Breeding pens | Mammalia | *Odocoileus virginianus* |
| *Anopheles crucians* | 10/13/22 | Gadsden | Preserve | Mammalia | *Odocoileus virginianus* |
| *Anopheles quadrimaculatus* | 6/29/24 | Gadsden | Breeding pens | Mammalia | *Odocoileus virginianus* |
| *Culex erraticus* | 7/8/23 | Gadsden | Barn pens | Mammalia | *Odocoileus virginianus* |
| *Culex erraticus* | 6/29/24 | Gadsden | Breeding pens | - | - |
| *Culex erraticus* | 7/8/23 | Gadsden | Barn pens | Mammalia | *Odocoileus virginianus* |
| *Culiseta melanura* | 10/13/22 | Gadsden | Preserve | Aves | *Hylocichla mustelina* |
| *Culex erraticus* | 7/4/23 | Gadsden | Breeding pens | - | - |
| *Culiseta melanura* | 10/13/22 | Gadsden | Preserve | - | - |
| *Culex erraticus* | 7/8/23 | Gadsden | Barn pens | Mammalia | *Odocoileus virginianus* |
| *Culiseta melanura* | 10/13/22 | Gadsden | Preserve | Aves | *Vireo griseus* |
| *Culex erraticus* | 7/8/23 | Gadsden | Barn pens | - | - |
| *Culiseta melanura* | 10/13/22 | Gadsden | Preserve | Aves | *Hylocichla mustelina* |
| *Culex erraticus* | 7/8/23 | Gadsden | Barn pens | - | - |
| *Culiseta melanura* | 10/13/22 | Gadsden | Preserve | Aves | *Hylocichla mustelina* |
| *Culiseta melanura* | 10/13/22 | Gadsden | Preserve | - | - |
| *Culex erraticus* | 7/8/23 | Gadsden | Barn pens | - | - |
| *Culex erraticus* | 7/4/23 | Gadsden | Breeding pens | - | - |
| *Culiseta melanura* | 10/13/22 | Gadsden | Preserve | - | - |
| *Culex erraticus* | 7/8/23 | Gadsden | Barn pens | - | - |
| *Culiseta melanura* | 10/13/22 | Gadsden | Preserve | Aves | *Hylocichla mustelina* |
| *Culex erraticus* | 7/8/23 | Gadsden | Barn pens | - | - |
| *Anopheles quadrimaculatus* | 10/13/22 | BT | NA | Aves | *Hylocichla mustelina* |
| *Aedes vexans* | 7/6/23 | Gadsden | Barn pens | - | - |
| *Culiseta melanura* | 10/13/22 | Gadsden | Preserve | Aves | *Thryothorus ludovicianus* |
| *Culiseta melanura* | 7/6/23 | Gadsden | Preserve | - | - |
| *Culiseta melanura* | 10/13/22 | Gadsden | Preserve | Aves | *Hylocichla mustelina* |
| *Culiseta melanura* | 10/13/22 | Gadsden | Preserve | - | - |
| *Aedes vexans* | 7/6/23 | Gadsden | Preserve | - | - |
| *Culiseta melanura* | 10/13/22 | Gadsden | Preserve | Aves | *Baeolophus atricristatus* |
| *Culiseta melanura* | 10/13/22 | Gadsden | Preserve | - | - |
| *Culex erraticus* | 7/6/23 | Gadsden | Barn pens | - | - |
| *Culiseta melanura* | 10/13/22 | Gadsden | Preserve | - | - |
| *Culex erraticus* | 8/16/23 | Gadsden | Barn pens | - | - |
| *Culex erraticus* | 8/16/23 | Gadsden | Barn pens | - | - |
| *Anopheles punctipennis* | 6/29/24 | Gadsden | Breeding pens | Mammalia | *Axis axis* |
| *Culiseta melanura* | 10/13/22 | Gadsden | Preserve | Mammalia | *Odocoileus virginianus* |
| *Culiseta melanura* | 10/13/22 | Gadsden | Breeding pens | - | - |
| *Culex erraticus* | 8/15/23 | Gadsden | Barn pens | - | - |
| *Anopheles punctipennis* | 6/29/24 | Gadsden | Breeding pens | - | - |
| *Culex erraticus* | 8/15/23 | Gadsden | Barn pens | - | - |
| *Culex erraticus* | 10/13/22 | Gadsden | Preserve | Mammalia | *Odocoileus virginianus* |
| *Culiseta melanura* | 5/20/23 | Gadsden | Preserve | Aves | *Strix varia* |
| *Culiseta melanura* | 5/20/23 | Gadsden | Preserve | - | - |
| *Culiseta melanura* | 5/19/23 | Gadsden | Preserve | - | - |
| *Anopheles punctipennis* | 7/8/23 | Gadsden | Breeding pens | Mammalia | *Odocoileus virginianus* |
| *Culiseta melanura* | 5/19/23 | Gadsden | Preserve | - | - |
| *Culex erraticus* | 5/20/23 | Gadsden | Breeding pens | - | - |
| *Culex erraticus* | 5/17/23 | Gadsden | Preserve | - | - |
| *Culex erraticus* | 8/16/23 | Gadsden | NA | Mammalia | *Odocoileus virginianus* |
| *Culex erraticus* | 5/17/23 | Gadsden | Preserve | - | - |
| *Culex erraticus* | 8/16/23 | Gadsden | NA | Mammalia | *Odocoileus virginianus* |
| *Culiseta melanura* | 5/20/23 | Gadsden | Preserve | - | - |
| *Culiseta melanura* | 5/17/23 | Gadsden | Preserve | - | - |
| *Culex erraticus* | 8/16/23 | Gadsden | NA | Mammalia | *Odocoileus virginianus* |
| *Culiseta melanura* | 5/17/23 | Gadsden | Preserve | - | - |
| *Culiseta melanura* | 5/17/23 | Gadsden | Preserve | - | - |
| *Culex erraticus* | 8/16/23 | Gadsden | NA | Mammalia | *Odocoileus virginianus* |
| *Culex erraticus* | 8/16/23 | Gadsden | NA | - | - |
| *Culiseta melanura* | 5/17/23 | Gadsden | Preserve | - | - |
| *Culex erraticus* | 5/16/23 | Gadsden | Preserve | - | - |
| *Culiseta melanura* | 5/19/23 | Gadsden | Barn pens | - | - |
| *Culiseta melanura* | 5/19/23 | Gadsden | Barn pens | - | - |
| *Culiseta melanura* | 5/20/23 | Gadsden | Preserve | - | - |
| *Culex erraticus* | 8/16/23 | Gadsden | NA | Mammalia | *Odocoileus virginianus* |
| *Culex erraticus* | 5/16/23 | Gadsden | Breeding pens | - | - |
| *Culex erraticus* | 5/16/23 | Gadsden | Breeding pens | - | - |
| *Culex erraticus* | 8/16/23 | Gadsden | NA | Mammalia | *Odocoileus virginianus* |
| *Culex erraticus* | 5/16/23 | Gadsden | Preserve | - | - |
| *Culex erraticus* | 5/16/23 | Gadsden | Preserve | - | - |
| *Culiseta melanura* | 5/16/23 | Gadsden | Barn pens | - | - |
| *Culex erraticus* | 8/16/23 | Gadsden | NA | Mammalia | *Odocoileus virginianus* |
| *Culiseta melanura* | 5/16/23 | Gadsden | Barn pens | - | - |
| *Culex erraticus* | 5/16/23 | Gadsden | Breeding pens | - | - |
| *Culex erraticus* | 5/16/23 | Gadsden | Breeding pens | - | - |
| *Culex erraticus* | 5/16/23 | Gadsden | Breeding pens | - | - |
| *Culex erraticus* | 5/20/23 | Gadsden | Barn pens | - | - |
| *Culiseta melanura* | 5/17/23 | Gadsden | Breeding pens | - | - |
| *Culex quinquefasciatus* | 8/14/23 | Gadsden | Barn pens | - | - |
| *Culex quinquefasciatus* | 5/17/23 | Gadsden | Barn pens | - | - |
| *Culex quinquefasciatus* | 8/14/23 | Gadsden | Barn pens | - | - |
| *Culex erraticus* | 5/17/23 | Gadsden | Barn pens | - | - |
| *Culex erraticus* | 8/14/23 | Gadsden | Barn pens | - | - |
| *Culex nigripalpus* | 5/16/23 | Gadsden | Preserve | - | - |
| *Culex erraticus* | 8/14/23 | Gadsden | Barn pens | - | - |
| *Anopheles crucians* | 6/29/24 | Gadsden | Preserve | Mammalia | *Odocoileus virginianus* |
| *Mansonia dyari* | 5/17/23 | Gadsden | Barn pens | - | - |
| *Culex erraticus* | 8/14/23 | Gadsden | Barn pens | - | - |
| *Anopheles crucians* | 6/27/24 | Gadsden | Barn pens | Mammalia | *Odocoileus virginianus* |
| *Culex erraticus* | 5/20/23 | Gadsden | Preserve | - | - |
| *Culiseta melanura* | 8/14/23 | Gadsden | Barn pens | - | - |
| *Anopheles crucians* | 6/27/24 | Gadsden | Barn pens | - | - |
| *Culex erraticus* | 8/14/23 | Gadsden | Barn pens | - | - |
| *Culex erraticus* | 8/14/23 | Gadsden | Barn pens | - | - |
| *Culex erraticus* | 8/14/23 | Gadsden | Preserve | - | - |
| *Culex erraticus* | 8/14/23 | Gadsden | Preserve | - | - |
| *Culiseta melanura* | 8/14/23 | Gadsden | Preserve | - | - |
| *Culiseta melanura* | 9/27/23 | Gadsden | Barn pens | - | - |
| *Aedes vexans* | 9/27/23 | Gadsden | Barn pens | - | - |
| *Culiseta melanura* | 9/28/23 | Gadsden | Breeding pens | - | - |
| *Culiseta melanura* | 9/28/23 | Gadsden | Breeding pens | - | - |
| *Culiseta melanura* | 9/28/23 | Gadsden | Breeding pens | - | - |
| *Anopheles crucians* | 9/28/23 | Gadsden | Barn pens | - | - |
| *Anopheles quadrimaculatus* | 7/6/23 | Gadsden | Breeding pens | Mammalia | *Odocoileus virginianus* |
| *Culiseta melanura* | 9/28/23 | Gadsden | Breeding pens | - | - |
| *Culiseta melanura* | 9/28/23 | Gadsden | Barn pens | - | - |
| *Culiseta melanura* | 9/28/23 | Gadsden | Barn pens | - | - |
| *Culiseta melanura* | 9/28/23 | Gadsden | Breeding pens | - | - |
| *Culiseta melanura* | 9/28/23 | Gadsden | Breeding pens | - | - |
| *Culiseta melanura* | 9/28/23 | Gadsden | Breeding pens | - | - |
| *Culex erraticus* | 9/28/23 | Gadsden | Preserve | - | - |
| *Culex erraticus* | 9/28/23 | Gadsden | Preserve | - | - |
| *Culex erraticus* | 9/29/23 | Gadsden | Barn pens | - | - |
| *Culiseta melanura* | 9/26/23 | Gadsden | Barn pens | - | - |
| *Culex erraticus* | 9/26/23 | Gadsden | Barn pens | - | - |
| *Culiseta melanura* | 9/26/23 | Gadsden | Barn pens | - | - |
| *Culiseta melanura* | 9/26/23 | Gadsden | Barn pens | - | - |
| *Culex erraticus* | 10/25/23 | Gadsden | Breeding pens | Mammalia | *Odocoileus virginianus* |
| *Anopheles crucians* | 6/29/24 | Gadsden | Barn pens | Mammalia | *Odocoileus virginianus* |
| *Culex erraticus* | 10/25/23 | Gadsden | Breeding pens | Mammalia | *Odocoileus virginianus* |
| *Culiseta melanura* | 10/25/23 | Gadsden | Preserve | - | - |
| *Anopheles crucians* | 6/29/24 | Gadsden | Barn pens | - | - |
| *Culiseta melanura* | 10/25/23 | Gadsden | Preserve | Mammalia | *Homo sapiens* |
| *Culiseta melanura* | 10/25/23 | Gadsden | Preserve | Mammalia | *Sylvilagus floridanus* |
| *Anopheles quadrimaculatus* | 6/28/24 | Gadsden | Preserve | Mammalia | *Odocoileus virginianus* |
| *Culiseta melanura* | 9/26/23 | Gadsden | Barn pens | Amphibia | *Anaxyrus terrestris* |
| *Culiseta melanura* | 9/26/23 | Gadsden | Barn pens | - | - |
| *Culex erraticus* | 9/26/23 | Gadsden | Barn pens | Mammalia | *Odocoileus virginianus* |
| *Culex erraticus* | 9/26/23 | Gadsden | Barn pens | Mammalia | *Homo sapiens* |
| *Culex erraticus* | 9/26/23 | Gadsden | Barn pens | Mammalia | *Sylvilagus floridanus* |
| *Anopheles crucians* | 9/26/23 | Gadsden | Barn pens | Mammalia | *Odocoileus virginianus* |
| *Culex nigripalpus* | 9/26/23 | Gadsden | Barn pens | Mammalia | *Odocoileus virginianus* |
| *Culiseta melanura* | 9/26/23 | Gadsden | Barn pens | - | - |
| *Culiseta melanura* | 9/26/23 | Gadsden | Barn pens | - | - |
| *Anopheles punctipennis* | 6/29/24 | Gadsden | Barn pens | - | - |
| *Culiseta melanura* | 9/26/23 | Gadsden | Barn pens | - | - |
| *Anopheles crucians* | 6/29/24 | Gadsden | Barn pens | - | - |
| *Culex peccator* | 9/26/23 | Gadsden | Barn pens | - | - |
| *Culex erraticus* | 9/26/23 | Gadsden | Barn pens | - | - |
| *Anopheles crucians* | 9/26/23 | Gadsden | Barn pens | - | - |
| *Culex erraticus* | 9/26/23 | Gadsden | Barn pens | - | - |
| *Culex erraticus* | 9/26/23 | Gadsden | Barn pens | - | - |
| *Anopheles crucians* | 6/29/24 | Gadsden | Barn pens | - | - |
| *Anopheles punctipennis* | 6/29/24 | Gadsden | Barn pens | - | - |
| *Culiseta melanura* | 9/26/23 | Gadsden | Barn pens | - | - |
| *Anopheles crucians* | 6/29/24 | Gadsden | Preserve | Mammalia | *Odocoileus virginianus* |
| *Culiseta melanura* | 9/26/23 | Gadsden | Preserve | - | - |
| *Culex erraticus* | 9/26/23 | Gadsden | Preserve | - | - |
| *Anopheles quadrimaculatus* | 6/25/24 | Gadsden | Breeding pens | Mammalia | *Sylvilagus floridanus* |
| *Anopheles crucians* | 9/26/23 | Gadsden | Preserve | - | - |
| *Culiseta melanura* | 9/26/23 | Gadsden | Preserve | - | - |
| *Psorophora ferox* | 9/26/23 | Gadsden | Preserve | - | - |
| *Culex erraticus* | 9/26/23 | Gadsden | Preserve | - | - |
| *Culiseta melanura* | 9/29/23 | Gadsden | Preserve | - | - |
| *Culiseta melanura* | 9/29/23 | Gadsden | Preserve | - | - |
| *Culiseta melanura* | 9/29/23 | Gadsden | Breeding pens | - | - |
| *Culex erraticus* | 9/29/23 | Gadsden | Barn pens | - | - |
| *Culiseta melanura* | 9/29/23 | Gadsden | Preserve | - | - |
| *Culiseta melanura* | 9/29/23 | Gadsden | Breeding pens | - | - |
| *Culiseta melanura* | 9/29/23 | Gadsden | Breeding pens | - | - |
| *Culiseta melanura* | 9/29/23 | Gadsden | Breeding pens | - | - |
| *Culiseta melanura* | 9/29/23 | Gadsden | Preserve | - | - |
| *Culiseta melanura* | 9/29/23 | Gadsden | Preserve | - | - |
| *Culiseta melanura* | 9/29/23 | Gadsden | Preserve | - | - |
| *Anopheles crucians* | 9/29/23 | Gadsden | Preserve | - | - |
| *Anopheles crucians* | 9/29/23 | Gadsden | Preserve | Mammalia | *Bos taurus* |
| *Culex erraticus* | 9/29/23 | Gadsden | Barn pens | - | - |
| *Culiseta melanura* | 9/30/23 | Gadsden | Breeding pens | - | - |
| *Culex erraticus* | 9/30/23 | Gadsden | Preserve | - | - |
| *Culiseta melanura* | 9/30/23 | Gadsden | Preserve | - | - |
| *Culex erraticus* | 9/30/23 | Gadsden | Breeding pens | - | - |
| *Culex erraticus* | 9/30/23 | Gadsden | Preserve | - | - |
| *Culex erraticus* | 9/30/23 | Gadsden | Preserve | - | - |
| *Culex erraticus* | 9/30/23 | Gadsden | Preserve | - | - |
| *Culex erraticus* | 8/16/23 | Gadsden | Preserve | - | - |
| *Culex erraticus* | 8/16/23 | Gadsden | Preserve | - | - |
| *Culex erraticus* | 8/16/23 | Gadsden | Preserve | - | - |
| *Culex erraticus* | 8/17/23 | Gadsden | Barn pens | - | - |
| *Culex erraticus* | 8/17/23 | Gadsden | Barn pens | - | - |
| *Culex erraticus* | 8/17/23 | Gadsden | Barn pens | - | - |
| *Culex erraticus* | 8/17/23 | Gadsden | Barn pens | - | - |
| *Culex erraticus* | 8/17/23 | Gadsden | Barn pens | - | - |
| *Culex erraticus* | 8/17/23 | Gadsden | Barn pens | - | - |
| *Culex erraticus* | 8/17/23 | Gadsden | Barn pens | - | - |
| *Culex erraticus* | 8/17/23 | Gadsden | Barn pens | - | - |
| *Culex erraticus* | 8/17/23 | Gadsden | Barn pens | - | - |
| *Culex erraticus* | 8/17/23 | Gadsden | Barn pens | - | - |
| *Culex erraticus* | 8/17/23 | Gadsden | Barn pens | - | - |
| *Culex erraticus* | 8/17/23 | Gadsden | Barn pens | - | - |
| *Culiseta melanura* | 8/17/23 | Gadsden | Barn pens | - | - |
| *Culex erraticus* | 8/17/23 | Gadsden | Barn pens | - | - |
| *Culex erraticus* | 8/17/23 | Gadsden | Barn pens | - | - |
| *Culex erraticus* | 8/17/23 | Gadsden | Barn pens | - | - |
| *Culex erraticus* | 8/17/23 | Gadsden | Barn pens | - | - |
| *Culex quinquefasciatus* | 8/17/23 | Gadsden | Barn pens | Mammalia | *Procyon lotor* |
| *Culex nigripalpus* | 8/16/23 | Gadsden | Barn pens | Aves | *Vireo olivaceus* |
| *Culex erraticus* | 8/17/23 | Gadsden | Barn pens | Mammalia | *Odocoileus virginianus* |
| *Culex erraticus* | 8/17/23 | Gadsden | Barn pens | Mammalia | *Odocoileus virginianus* |
| *Culex erraticus* | 8/17/23 | Gadsden | Barn pens | Mammalia | *Odocoileus virginianus* |
| *Culiseta melanura* | 8/17/23 | Gadsden | Preserve | Mammalia | *Odocoileus virginianus* |
| *Culiseta melanura* | 8/17/23 | Gadsden | Preserve | Aves | *Coccyzus americanus* |
| *Culiseta melanura* | 8/17/23 | Gadsden | Preserve | Aves | *Cathartes aura* |
| *Culiseta melanura* | 8/17/23 | Gadsden | Preserve | Aves | *Poecile atricapilla* |
| *Culiseta melanura* | 8/17/23 | Gadsden | Preserve | - | - |
| *Culex erraticus* | 8/17/23 | Gadsden | Breeding pens | - | - |
| *Culex erraticus* | 8/17/23 | Gadsden | Breeding pens | Mammalia | *Odocoileus virginianus* |
| *Culiseta melanura* | 8/17/23 | Gadsden | Breeding pens | Aves | *Cardinalis cardinalis* |
| *Culex erraticus* | 8/17/23 | Gadsden | Breeding pens | Mammalia | *Odocoileus virginianus* |
| *Culex erraticus* | 8/17/23 | Gadsden | Breeding pens | Mammalia | *Odocoileus virginianus* |
| *Culex erraticus* | 8/17/23 | Gadsden | Breeding pens | Mammalia | *Odocoileus virginianus* |
| *Culiseta melanura* | 8/17/23 | Gadsden | Breeding pens | Mammalia | *Odocoileus virginianus* |
| *Culiseta melanura* | 8/17/23 | Gadsden | Breeding pens | Mammalia | *Odocoileus virginianus* |
| *Culiseta melanura* | 8/15/23 | Gadsden | Breeding pens | - | - |
| *Culiseta melanura* | 8/15/23 | Gadsden | Breeding pens | - | - |
| *Culiseta melanura* | 8/15/23 | Gadsden | Breeding pens | Aves | *Cardinalis cardinalis* |
| *Culex erraticus* | 8/15/23 | Gadsden | Breeding pens | Reptilia | *Anolis carolinensis* |
| *Anopheles crucians* | 8/17/23 | Gadsden | Breeding pens | Aves | *Cardinalis cardinalis* |
| *Culex erraticus* | 8/17/23 | Gadsden | Barn pens | Aves | *Meleagris gallopavo* |
| *Culex erraticus* | 8/17/23 | Gadsden | Barn pens | Reptilia | *Anolis carolinensis* |
| *Culex erraticus* | 8/17/23 | Gadsden | Barn pens | Mammalia | *Odocoileus virginianus* |
| *Culex erraticus* | 8/15/23 | Gadsden | Barn pens | - | - |
| *Culex erraticus* | 8/15/23 | Gadsden | Barn pens | - | - |
| *Culex erraticus* | 8/15/23 | Gadsden | Barn pens | Mammalia | *Odocoileus virginianus* |
| *Culex erraticus* | 8/15/23 | Gadsden | Barn pens | Mammalia | *Odocoileus virginianus* |
| *Culex erraticus* | 8/15/23 | Gadsden | Barn pens | Mammalia | *Odocoileus virginianus* |
| *Culex erraticus* | 8/15/23 | Gadsden | Barn pens | Mammalia | *Odocoileus virginianus* |
| *Culex erraticus* | 8/15/23 | Gadsden | Barn pens | Mammalia | *Odocoileus virginianus* |
| *Culex erraticus* | 8/15/23 | Gadsden | Barn pens | Mammalia | *Odocoileus virginianus* |
| *Culex erraticus* | 8/15/23 | Gadsden | Barn pens | Mammalia | *Odocoileus virginianus* |
| *Culex erraticus* | 8/15/23 | Gadsden | Barn pens | Mammalia | *Odocoileus virginianus* |
| *Culex erraticus* | 8/15/23 | Gadsden | Barn pens | Mammalia | *Odocoileus virginianus* |
| *Culex erraticus* | 8/15/23 | Gadsden | Barn pens | - | - |
| *Culex erraticus* | 8/15/23 | Gadsden | Barn pens | Mammalia | *Odocoileus virginianus* |
| *Culex erraticus* | 8/15/23 | Gadsden | Barn pens | - | - |
| *Culex erraticus* | 8/15/23 | Gadsden | Barn pens | - | - |
| *Culex erraticus* | 8/15/23 | Gadsden | Barn pens | Mammalia | *Odocoileus virginianus* |
| *Culex erraticus* | 8/15/23 | Gadsden | Barn pens | - | - |
| *Culex erraticus* | 8/15/23 | Gadsden | Barn pens | Mammalia | *Odocoileus virginianus* |
| *Culex erraticus* | 8/15/23 | Gadsden | Barn pens | Mammalia | *Odocoileus virginianus* |
| *Culex erraticus* | 8/15/23 | Gadsden | Barn pens | Mammalia | *Odocoileus virginianus* |
| *Culex erraticus* | 8/15/23 | Gadsden | Barn pens | - | - |
| *Culiseta melanura* | 8/15/23 | Gadsden | Barn pens | - | - |
| *Culiseta melanura* | 8/15/23 | Gadsden | Barn pens | - | - |
| *Culiseta melanura* | 9/26/23 | Gadsden | Barn pens | - | - |
| *Culiseta melanura* | 9/26/23 | Gadsden | Barn pens | - | - |
| *Culiseta melanura* | 9/26/23 | Gadsden | Barn pens | - | - |
| *Culiseta melanura* | 9/26/23 | Gadsden | Barn pens | - | - |
| *Culex erraticus* | 9/26/23 | Gadsden | Breeding pens | Mammalia | *Odocoileus virginianus* |
| *Culiseta melanura* | 9/26/23 | Gadsden | Breeding pens | - | - |
| *Culiseta melanura* | 9/26/23 | Gadsden | Breeding pens | Aves | *Vireo olivaceus* |
| *Anopheles crucians* | 9/26/23 | Gadsden | Breeding pens | Mammalia | *Odocoileus virginianus* |
| *Culiseta melanura* | 9/26/23 | Gadsden | Breeding pens | Aves | *Catharus fuscescens* |
| *Culex erraticus* | 9/26/23 | Gadsden | Breeding pens | Mammalia | *Odocoileus virginianus* |
| *Culex erraticus* | 9/26/23 | Gadsden | Breeding pens | Mammalia | *Ursus americanus* |
| *Culex erraticus* | 9/26/23 | Gadsden | Breeding pens | Aves | *Catharus fuscescens* |
| *Culiseta melanura* | 9/26/23 | Gadsden | Breeding pens | Mammalia | *Mus musculus* |
| *Culex erraticus* | 9/26/23 | Gadsden | Barn pens | Mammalia | *Odocoileus virginianus* |
| *Culiseta melanura* | 9/26/23 | Gadsden | Breeding pens | - | - |
| *Culiseta melanura* | 9/26/23 | Gadsden | Breeding pens | - | - |
| *Culiseta melanura* | 9/26/23 | Gadsden | Breeding pens | - | - |
| *Culiseta melanura* | 9/27/23 | Gadsden | Barn pens | - | - |
| *Culiseta melanura* | 9/27/23 | Gadsden | Preserve | - | - |
| *Culiseta melanura* | 9/27/23 | Gadsden | Preserve | - | - |
| *Culex erraticus* | 9/27/23 | Gadsden | Barn pens | Mammalia | *Odocoileus virginianus* |
| *Culiseta melanura* | 9/27/23 | Gadsden | Breeding pens | - | - |
| *Culex erraticus* | 9/27/23 | Gadsden | Barn pens | - | - |
| *Culiseta melanura* | 9/26/23 | Gadsden | Preserve | - | - |
| *Culiseta melanura* | 9/27/23 | Gadsden | Breeding pens | - | - |
| *Culiseta melanura* | 9/27/23 | Gadsden | Breeding pens | - | - |
| *Culiseta melanura* | 9/27/23 | Gadsden | Preserve | - | - |
| *Culiseta melanura* | 9/27/23 | Gadsden | Barn pens | - | - |
| *Culiseta melanura* | 9/27/23 | Gadsden | Breeding pens | - | - |
| *Culex erraticus* | 9/27/23 | Gadsden | Barn pens | Mammalia | *Odocoileus virginianus* |
| *Culiseta melanura* | 9/27/23 | Gadsden | Barn pens | - | - |
| *Anopheles crucians* | 9/27/23 | Gadsden | Breeding pens | Mammalia | *Odocoileus virginianus* |
| *Aedes vexans* | 9/27/23 | Gadsden | Barn pens | - | - |
| *Culiseta melanura* | 9/27/23 | Gadsden | Barn pens | Mammalia | *Procyon lotor* |
| *Culiseta melanura* | 9/27/23 | Gadsden | Barn pens | - | - |
| *Culiseta melanura* | 9/27/23 | Gadsden | Barn pens | - | - |
| *Culiseta melanura* | 9/27/23 | Gadsden | Barn pens | Mammalia | *Marmota flaviventris* |
| *Culiseta melanura* | 9/27/23 | Gadsden | Barn pens | Mammalia | *Odocoileus virginianus* |
| *Culiseta melanura* | 9/27/23 | Gadsden | Barn pens | Mammalia | *Odocoileus virginianus* |
| *Culex erraticus* | 9/27/23 | Gadsden | Barn pens | Mammalia | *Odocoileus virginianus* |
| *Aedes vexans* | 9/27/23 | Gadsden | Breeding pens | Mammalia | *Odocoileus virginianus* |
| *Culex erraticus* | 9/29/23 | Gadsden | Breeding pens | Mammalia | *Odocoileus virginianus* |
| *Culiseta melanura* | 9/29/23 | Gadsden | Breeding pens | - | - |
| *Anopheles punctipennis* | 9/29/23 | Gadsden | Preserve | Mammalia | *Sylvilagus floridanus* |
| *Anopheles punctipennis* | 9/29/23 | Gadsden | Preserve | Mammalia | *Dama dama* |
| *Anopheles crucians* | 9/29/23 | Gadsden | Barn pens | Mammalia | *Odocoileus virginianus* |
| *Anopheles quadrimaculatus* | 9/30/23 | Gadsden | Preserve | Mammalia | *Odocoileus virginianus* |
| *Culiseta melanura* | 9/30/23 | Gadsden | Barn pens | Mammalia | *Homo sapiens* |
| *Anopheles punctipennis* | 9/29/23 | Gadsden | Barn pens | Mammalia | *Odocoileus virginianus* |
| *Anopheles punctipennis* | 9/29/23 | Gadsden | Barn pens | Mammalia | *Odocoileus virginianus* |
| *Anopheles crucians* | 8/14/23 | Gadsden | Barn pens | Mammalia | *Odocoileus virginianus* |
| *Anopheles punctipennis* | 10/26/23 | Gadsden | Barn pens | Mammalia | *Odocoileus virginianus* |
| *Anopheles punctipennis* | 10/26/23 | Gadsden | Breeding pens | Mammalia | *Odocoileus virginianus* |
| *Anopheles punctipennis* | 10/26/23 | Gadsden | Breeding pens | Mammalia | *Odocoileus virginianus* |
| *Anopheles crucians* | 5/13/24 | Gadsden | Breeding pens | Mammalia | *Odocoileus virginianus* |
| *Anopheles punctipennis* | 5/13/24 | Gadsden | Breeding pens | - | - |
| *Anopheles crucians* | 5/14/24 | Gadsden | Breeding pens | Mammalia | *Odocoileus virginianus* |
| *Anopheles crucians* | 5/15/24 | Gadsden | Breeding pens | Mammalia | *Odocoileus virginianus* |
| *Anopheles quadrimaculatus* | 5/15/24 | Gadsden | Breeding pens | Mammalia | *Odocoileus virginianus* |
| *Anopheles crucians* | 5/15/24 | Gadsden | Breeding pens | Mammalia | *Odocoileus virginianus* |
| *Anopheles crucians* | 5/15/24 | Gadsden | Breeding pens | Mammalia | *Odocoileus virginianus* |
| *Anopheles crucians* | 5/15/24 | Gadsden | Breeding pens | Mammalia | *Odocoileus virginianus* |
| *Anopheles crucians* | 5/15/24 | Gadsden | Breeding pens | Mammalia | *Odocoileus virginianus* |
| *Anopheles crucians* | 5/14/24 | Gadsden | Breeding pens | Mammalia | *Odocoileus virginianus* |
| *Anopheles punctipennis* | 5/15/24 | Gadsden | Breeding pens | Mammalia | *Odocoileus virginianus* |
| *Anopheles punctipennis* | 5/15/24 | Gadsden | Breeding pens | Mammalia | *Odocoileus virginianus* |
| *Anopheles quadrimaculatus* | 5/15/24 | Gadsden | Breeding pens | Mammalia | *Odocoileus virginianus* |
| *Anopheles quadrimaculatus* | 5/15/24 | Gadsden | Breeding pens | Mammalia | *Odocoileus virginianus* |
| *Anopheles punctipennis* | 5/14/24 | Gadsden | Breeding pens | Mammalia | *Odocoileus virginianus* |
| *Anopheles punctipennis* | 5/14/24 | Gadsden | Breeding pens | Mammalia | *Odocoileus virginianus* |
| *Anopheles crucians* | 5/14/24 | Gadsden | Breeding pens | - | - |
| *Anopheles punctipennis* | 5/14/24 | Gadsden | Breeding pens | Mammalia | *Odocoileus virginianus* |
| *Anopheles crucians* | 5/14/24 | Gadsden | Breeding pens | Mammalia | *Odocoileus virginianus* |
| *Anopheles crucians* | 5/14/24 | Gadsden | Breeding pens | Mammalia | *Odocoileus virginianus* |
| *Anopheles crucians* | 5/14/24 | Gadsden | Breeding pens | Reptilia | *Anolis carolinensis* |
| *Anopheles crucians* | 5/14/24 | Gadsden | Breeding pens | - | - |
| *Anopheles punctipennis* | 5/15/24 | Gadsden | NA | Mammalia | *Odocoileus virginianus* |
| *Anopheles punctipennis* | 5/15/24 | Gadsden | Breeding pens | Mammalia | *Odocoileus virginianus* |
| *Anopheles crucians* | 5/15/24 | Gadsden | Breeding pens | Mammalia | *Odocoileus virginianus* |
| *Anopheles punctipennis* | 5/14/24 | Gadsden | Breeding pens | Mammalia | *Odocoileus virginianus* |
| *Anopheles punctipennis* | 5/14/24 | Gadsden | Breeding pens | Mammalia | *Odocoileus virginianus* |
| *Anopheles punctipennis* | 5/15/24 | Gadsden | Breeding pens | Mammalia | *Odocoileus virginianus* |
| *Anopheles crucians* | 5/15/24 | Gadsden | Breeding pens | - | - |
| *Anopheles crucians* | 5/17/24 | Gadsden | Preserve | Mammalia | *Odocoileus virginianus* |
| *Anopheles punctipennis* | 5/17/24 | Gadsden | Barn pens | Mammalia | *Odocoileus virginianus* |
| *Anopheles punctipennis* | 5/19/24 | Gadsden | Breeding pens | Mammalia | *Odocoileus virginianus* |
| *Anopheles crucians* | 5/17/24 | Gadsden | Preserve | Mammalia | *Odocoileus virginianus* |
| *Anopheles crucians* | 5/17/24 | Gadsden | Preserve | Mammalia | *Odocoileus virginianus* |
| *Anopheles crucians* | 5/14/24 | Gadsden | Breeding pens | Mammalia | *Odocoileus virginianus* |
| *Anopheles punctipennis* | 5/17/24 | Gadsden | Preserve | - | - |
| *Anopheles punctipennis* | 5/17/24 | Gadsden | Breeding pens | Mammalia | *Odocoileus virginianus* |
| *Anopheles crucians* | 5/17/24 | Gadsden | Breeding pens | - | - |
| *Anopheles quadrimaculatus* | 5/15/24 | Gadsden | Preserve | - | - |
| *Anopheles crucians* | 5/15/24 | Gadsden | Preserve | Mammalia | *Odocoileus virginianus* |
| *Anopheles crucians* | 5/15/24 | Gadsden | Preserve | - | - |
| *Anopheles crucians* | 5/15/24 | Gadsden | Preserve | Mammalia | *Bos taurus* |
| *Anopheles crucians* | 5/15/24 | Gadsden | Preserve | Mammalia | *Odocoileus virginianus* |
| *Anopheles quadrimaculatus* | 5/16/24 | Gadsden | Breeding pens | Mammalia | *Odocoileus virginianus* |
| *Anopheles quadrimaculatus* | 5/16/24 | Gadsden | Breeding pens | Mammalia | *Odocoileus virginianus* |
| *Anopheles crucians* | 5/16/24 | Gadsden | Breeding pens | Mammalia | *Odocoileus virginianus* |
| *Anopheles crucians* | 5/16/24 | Gadsden | Breeding pens | Mammalia | *Odocoileus virginianus* |
| *Anopheles crucians* | 5/16/24 | Gadsden | Breeding pens | Mammalia | *Odocoileus virginianus* |
| *Anopheles crucians* | 5/16/24 | Gadsden | Breeding pens | - | - |
| *Anopheles crucians* | 5/16/24 | Gadsden | Breeding pens | - | - |
| *Anopheles crucians* | 5/16/24 | Gadsden | Breeding pens | Mammalia | *Odocoileus virginianus* |
| *Anopheles crucians* | 5/16/24 | Gadsden | Breeding pens | Mammalia | *Odocoileus virginianus* |
| *Anopheles quadrimaculatus* | 5/18/24 | Gadsden | Preserve | - | - |
| *Anopheles crucians* | 5/18/24 | Gadsden | Breeding pens | Mammalia | *Sylvilagus floridanus* |
| *Anopheles punctipennis* | 5/13/24 | Gadsden | NA | Mammalia | *Odocoileus virginianus* |
| *Anopheles crucians* | 5/19/24 | Gadsden | Barn pens | - | - |
| *Anopheles crucians* | 5/19/24 | Gadsden | Breeding pens | - | - |
| *Anopheles crucians* | 5/19/24 | Gadsden | Breeding pens | Mammalia | *Odocoileus virginianus* |
| *Anopheles crucians* | 5/19/24 | Gadsden | Breeding pens | - | - |
| *Anopheles punctipennis* | 5/17/24 | Gadsden | Breeding pens | Mammalia | *Odocoileus virginianus* |
| *Anopheles crucians* | 5/17/24 | Gadsden | Breeding pens | Mammalia | *Antilope cervicapra* |
| *Anopheles crucians* | 5/15/24 | Gadsden | Breeding pens | - | - |
| *Anopheles crucians* | 5/19/24 | Gadsden | Breeding pens | Reptilia | *Anolis carolinensis* |
| *Anopheles crucians* | 5/18/24 | Gadsden | Preserve | - | - |
| *Anopheles crucians* | 5/15/24 | Gadsden | Breeding pens | Reptilia | *Anolis carolinensis* |
| *Anopheles punctipennis* | 6/28/24 | Gadsden | Breeding pens | Reptilia | *Anolis carolinensis* |
